# Global Genome Mining Reveals the Distribution of Diverse Thioamidated RiPP Biosynthesis Gene Clusters

**DOI:** 10.3389/fmicb.2021.635389

**Published:** 2021-04-30

**Authors:** Jessie James Limlingan Malit, Chuanhai Wu, Ling-Li Liu, Pei-Yuan Qian

**Affiliations:** ^1^Department of Ocean Science and Hong Kong Branch of Southern Marine Science and Engineering Guangdong Laboratory, The Hong Kong University of Science and Technology, Hong Kong, China; ^2^Shaanxi Key Laboratory of Natural Products and Chemical Biology, College of Chemistry and Pharmacy, Northwest A&F University, Yangling, China

**Keywords:** TfuA, RiPPs, genome mining, YcaO, biosynthesis pathway, thioamide

## Abstract

Thioamidated ribosomally synthesized and post-translationally modified peptides (RiPPs) are recently characterized natural products with wide range of potent bioactivities, such as antibiotic, antiproliferative, and cytotoxic activities. These peptides are distinguished by the presence of thioamide bonds in the peptide backbone catalyzed by the YcaO-TfuA protein pair with its genes adjacent to each other. Genome mining has facilitated an *in silico* approach to identify biosynthesis gene clusters (BGCs) responsible for thioamidated RiPP production. In this work, publicly available genomic data was used to detect and illustrate the diversity of putative BGCs encoding for thioamidated RiPPs. AntiSMASH and RiPPER analysis identified 613 unique TfuA-related gene cluster families (GCFs) and 797 precursor peptide families, even on phyla where the presence of these clusters have not been previously described. Several additional biosynthesis genes are colocalized with the detected BGCs, suggesting an array of possible chemical modifications. This study shows that thioamidated RiPPs occupy a widely unexplored chemical landscape.

## Introduction

Natural products belonging to the classes of ribosomally synthesized and post-translationally modified peptides (RiPPs) constitute one of the major sources of bioactive compounds (Mohimani et al., 2014). Their diverse chemical structures and therapeutic capacities (Skinnider et al., 2016) have garnered attention, especially their potential use to treat deadly infections caused by antimicrobial-resistant bacteria (Letzel et al., 2014). RiPPs are often produced initially as precursor peptides containing a core peptide that is flanked by either a leader or a follower peptide, which is recognized by modifying and transport enzymes (Arnison et al., 2013). Additional biosynthetic enzymes termed as RiPP tailoring enzymes (RTEs), which are found in proximity to the locus of the precursor peptide in the biosynthesis gene cluster (BGC), can structurally modify the core peptide and lead to the biosynthesis of highly modified products. RiPPs are divided into classes depending on the posttranslational modifications applied by these RTEs (Hetrick and van der Donk, 2017).

In contrast with non-ribosomal peptides (NRPs) and other classes of natural products, the ribosomal origin of RiPP precursors allows the use of genomic data for the reliable prediction of their preliminary chemical structure (Zhang et al., 2018). Irrespective of phyla (Ortega and van der Donk, 2016) and the conserved gene content and structure of their BGCs (Letzel et al., 2014), the common biosynthetic pathways for the production of each RiPP class have helped in accurately identifying RiPP BGCs from genomes. Global genome mining is an alternative way to identify specific BGCs from massive genomic data. Our group has recently developed a pipeline to discover novel gene clusters from global genome data (Li et al., 2018). Similar to the success in lassopeptides (Tietz et al., 2017), lanthipeptides (Walker et al., 2020), and thiopeptides (Schwalen et al., 2018), large-scale genomic analysis of bacterial genomes has enabled the representation of the massive chemical diversity of RiPPs.

**FIGURE 1:**
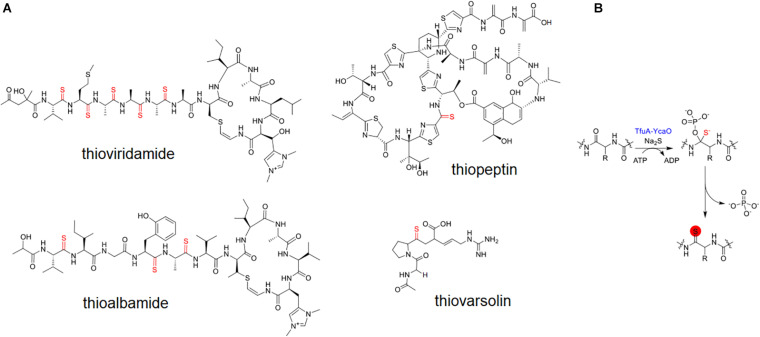
Known thioamidated RiPPs **(A)** and enzymatic mechanism for thioamidation **(B)**.

Thioamidated RiPPs are an interesting class of natural products characterized by incorporating sulfur instead of carbonyl oxygens in one or several peptide bonds (Kjaerulff et al., 2017). This modification imparts several pharmacological advantages for the compound by improving its physical stability (Reiner et al., 2008) and absorption, distribution, metabolism, and excretion (ADME) properties (Banala and Süssmuth, 2010). Only a handful of bacterial thioamidated RiPPs, such as thioviridamides (Hayakawa et al., 2006b) and their derivatives (Izumikawa et al., 2015; Tang et al., 2018), methanobactin (Kenney et al., 2018), thioholgamide (Kjaerulff et al., 2017), thioalbamide (Frattaruolo et al., 2019), thiostreptamide (Frattaruolo et al., 2017), thiopeptin (Liu et al., 2019) and thiovarsolins (Santos-Aberturas et al., 2019; Figure 1A), have been described and shown to display potent antibacterial (Kjaerulff et al., 2017) and antitumor activities (Hayakawa et al., 2006a). These bioactivities warrant the further exploration of these compounds as a potential new source of pharmaceutical drugs.

The biosynthesis gene responsible for the production of thioamidated ribosomal peptides have been recently identified (Burkhart et al., 2017; Santos-Aberturas et al., 2019). Following the elucidation of the thioviridamide BGC (Izawa et al., 2013), and the *in vitro* reconstitution of peptidic thioamidation in methanogenic archaea (Mahanta et al., 2018), two proteins with their coding genes adjacent, TfuA and YcaO, were found to directly catalyze the formation of thioamides on a precursor peptide (Burkhart et al., 2017). Thioamidation is catalyzed by YcaO through an ATP-dependent phosphorylation/adenylation mechanism that primarily involves a nucleophilic attack by sulfide on the peptidic amide bond, while TfuA is hypothesized to allosterically activate YcaO or aid in initial sulfidation (Figure 1B; Mahanta et al., 2018). A new genome mining platform RiPPER that identifies RIPP precursor peptides regardless of RiPP family was devised by Santos-Aberturas et al., and was applied to BGCs from Actinobacteria containing the two core enzymes associated with thioamidated RiPPs. This work led to the discovery of thiovarsolins from *Streptomyces varsoviensis* (Santos-Aberturas et al., 2019). Although methanobactin possesses thioamide bonds in its backbone, its biosynthesis does not involve TfuA-YcaO but of two hypothetical proteins MbnBC (Kenney et al., 2018), showing that thioamidation on peptides can be catalyzed by a different enzymatic route.

A global genome mining approach using antiSMASH (Blin et al., 2019) was applied on all available genomes to select BGCs containing adjacent YcaO and TfuA-like proteins to further depict the diversity of putative thioamidated RiPPs produced by bacteria. Neighboring precursor peptides that are possibly acted upon by these proteins were identified using RiPPER (Santos-Aberturas et al., 2019). Sequence similarity networking using BiG-SCAPE was performed to group similar BGCs together and to chart the diversity of the genetic architectures displayed by thioamidated RiPP BGCs. Several BGCs sharing similarities with characterized RiPPs and those that possess additional RTEs were also characterized. Motif discovery was conducted to identify sequence motifs specific to TfuA-associated YcaO.

## Materials and Methods

### Global Genomic Data

Annotated RefSeq genomes of all assembly levels (162,672) spanning the entire bacterial and archaeal kingdom were obtained (April 2020) from the National Center for Biotechnology Information (Kitts et al., 2016; Supplementary Tables 1, 2).

### Genome Mining for Thioamidated RiPPs

Genomes were analyzed using antiSMASH v5.1.2 (Blin et al., 2019) to identify the BGCs containing YcaO and TfuA-like proteins by employing profile HMMs. TfuA protein sequences were extracted and clustered using cd-hit (Fu et al., 2012) set at 100% similarity to account for repetitively sequenced and highly similar genomes. BGCs containing unique *tfuA* sequences were used for downstream analyses.

### BGC and Precursor Peptide Similarity Network Analysis

BGC similarity network from antiSMASH annotated files was generated by BiG-SCAPE (Navarro-Muñoz et al., 2020) with a multiple raw distance cutoff value c = 0.5. Precursor peptides encoded in the filtered BGCs were identified using RiPPER at standard settings (Santos-Aberturas et al., 2019), and the corresponding similarity network was then generated using EGN (Halary et al., 2013). Precursor peptide sequences were aligned using Clustal Omega (Sievers and Higgins, 2018), and sequence logos were generated using Weblogo (Crooks et al., 2004). TfuA protein sequence similarity network was generated using Enzyme Function Initiative-Enzyme Similarity Tool using an alignment score of 35 (Gerlt et al., 2015). All networks were visualized using Cytoscape 3.7.2 (Shannon et al., 2003).

### Phylogenetic Analysis and Motif Discovery

Protein sequences coding for TfuA-like proteins were retrieved from the filtered BGCs and were aligned using Clustal Omega (Sievers and Higgins, 2018). An approximated maximum likelihood phylogenetic tree was generated and visualized through FastTree (Price et al., 2010) and interactive Tree Of Life (iTOL) (Letunic and Bork, 2016), respectively. Translated protein sequences of *tfuA*-associated *ycaO* genes obtained from the BGCs detected in this study and non-*tfuA* associated *ycaO* genes extracted from the MiBIG database (Kautsar et al., 2020) were aligned using Clustal Omega (Sievers and Higgins, 2018). Protein sequence motifs were identified using MEME (Bailey et al., 2009) and were represented through sequence logos generated by Weblogo (Crooks et al., 2004).

## Results and Discussion

### AntiSMASH Analysis Shows Numerous Unidentified BGCs Encoding Putative Thioamidated RiPPs

**FIGURE 2:**
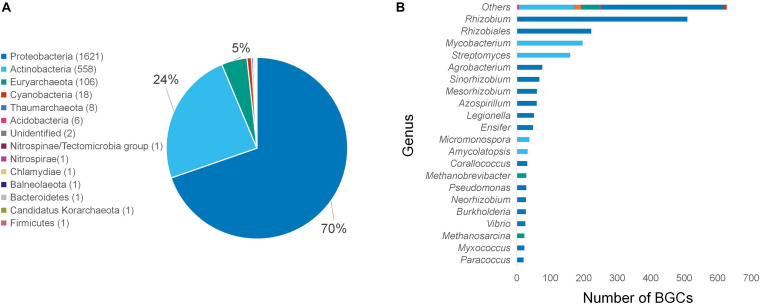
Number of unique thioamidated RiPP BGCs identified using antiSMASH organized per phylum **(A)** and genus **(B)**. Genera with less than 10 clusters identified were grouped into “Other.”

AntiSMASH uses rule-based detections derived from profile HMMs to identify conserved core enzymes and classify them into BGCs by using validated gene cluster rules (Blin et al., 2019). Only BGCs containing *ycaO* and *tfuA*-like genes, which are classified by antiSMASH as “TfuA-related,” were selected to categorize for BGCs putatively coding for thioamidated RiPPs. These BGCs were identified from 161,733 bacterial genomes and 939 archaeal genomes. After the removal of redundant sequences, the 14,520 classified putative thioamidated RiPP-encoding clusters were further reduced to 2,326 clusters (Supplementary Table 3). The majority of these unique, filtered clusters belong to the phylum Proteobacteria (70%) and Actinobacteria (24%). Several clusters from other phyla including Cyanobacteria and Acidobacteria were also identified. The wide distribution of phyla and genera reveals the relative ubiquity of these clusters in the bacterial kingdom (Figure 2A). Over 500 BGCs belonged to *Rhizobium*, a genus of Gram-negative soil bacteria that is known for nitrogen fixation (Figure 2B).

All of the currently known thioamidated RiPPs biosynthesized by the TfuA-YcaO protein pair are obtained from Actinobacteria. For the first time, this study found over a thousand thioamidated peptide-encoding BGC clusters belonging to Proteobacteria, a major phylum of Gram-negative bacteria that includes a wide variety of pathogenic genera. Although this result can be due to the overwhelming amount of sequenced proteobacterial species available online compared with other phyla, BGC sequence similarity network analysis still suggests that this phylum displays diverse BGC gene architectures, some of which have previously undefined chemical novelty. Recent comprehensive research work indicated that Gram-negative bacteria could be a rich underexplored source of novel antibiotics (Masschelein et al., 2017). Only one cluster was identified from Firmicutes, although this phylum was known to have the most number of RiPP BGCs encoded in their genomes (Skinnider et al., 2016). Several genomes originating from different phyla harbor more than one TfuA-cluster (Supplementary Table 4), with *Mycobacterium szulgai* DSM 44166 and *Mycobacterium angelicum* DSM 45057 having the most per genome with six clusters each.

Analysis of 939 archaeal genomes revealed 130 unique TfuA-related BGCs, which account for 5% of the total detected BGCs. Most clusters (106) belong to Euryarchaeota, which represents the third phylum with the most clusters, in agreement with a previous study (Mahanta et al., 2018). Eight BGCs were detected from Thaumarchaeota, another archaeal phylum, signifying the possible similar capability of its members to catalyze the same reaction. Although thioamidation by archaeal species has only been reported on methyl-coenzyme M reductase (MCR) (Mahanta et al., 2018) but not on RiPPs, the archaeal YcaO-TfuA pair was discovered to work on small peptides such as the small fragments of MCR (Mahanta et al., 2018). This finding implicates the possible diversification of small peptidic natural products through combinatorial biosynthesis and refactoring.

Cyanobacteria show the potential to produce a wide variety of bioactive compounds (Singh et al., 2005). Genome mining analysis identified 18 unique TfuA-related BGCs from Nostocales, Oscillatoriophycideae, and Gloeobacteria (Supplementary Table 3). This work is the first to reveal the genetic potential of Cyanobacteria to produce thioamidated compounds, which is worthy of further exploration.

### Sequence Similarity Network Analysis of TfuA-Related BGCs Identified by antiSMASH

**FIGURE 3:**
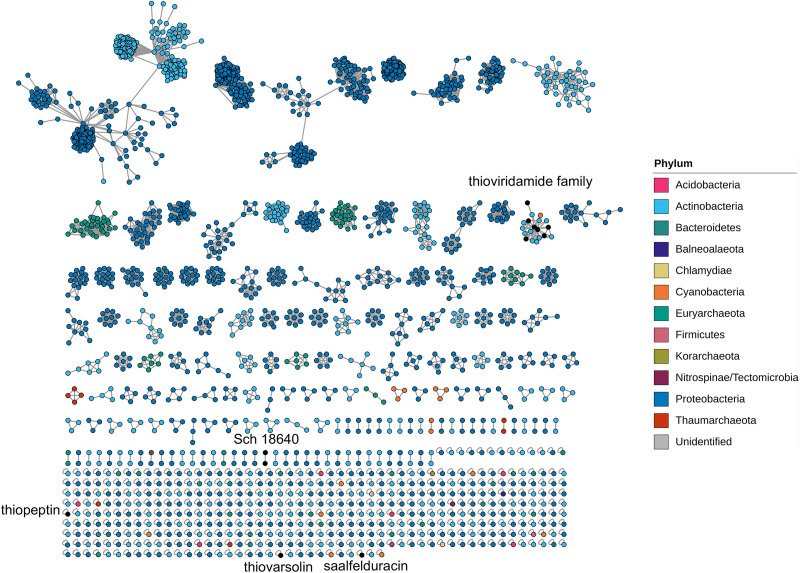
Sequence similarity network generated by BiG-SCAPE of TfuA-related BGCs identified by antiSMASH. The color of the node outline corresponds to the phylum of the organism harboring the biosynthesis gene cluster.

Biosynthetic Genes Similarity Clustering and Prospecting Engine (BiG-SCAPE) was used to chart the assortment of the genomic architecture of the TfuA-related BGCs. This tool creates a sequence similarity network (SSN) and groups similar BGCs into gene cluster families (GCFs) to map their diversity and evolution (Navarro-Muñoz et al., 2020). The generated SSN clearly confirms the diversity of the TfuA-containing BGCs as indicated by 613 distinct GCFs, 445 of which are singletons (Figure 3). More than half (59.7%) of the detected BGCs belong to Proteobacteria, which is found in 103 discrete GCFs and 263 singleton BGCs. On the other hand, 50 GCFs and 140 singletons are formed by actinobacterial species. Together, 190 unique representative BGCs are extracted from Actinobacteria. A previous RiPPER search for TfuA-like proteins in Actinobacteria yielded 225 clusters (Santos-Aberturas et al., 2019). The lesser number of BGCs detected in this study could be due to a higher raw distance cutoff used in grouping BGCs. BGCs belonging to the same taxonomic phylum are clustered exclusively, and several additional genes in the neighborhood of the *tfuA-ycaO* gene pair are conserved. Only nine BGCs exhibit similarity with known thioamidated RiPP BGCs, implying the widely thioamidated RiPP chemical space that is yet to be described.

**FIGURE 4:**
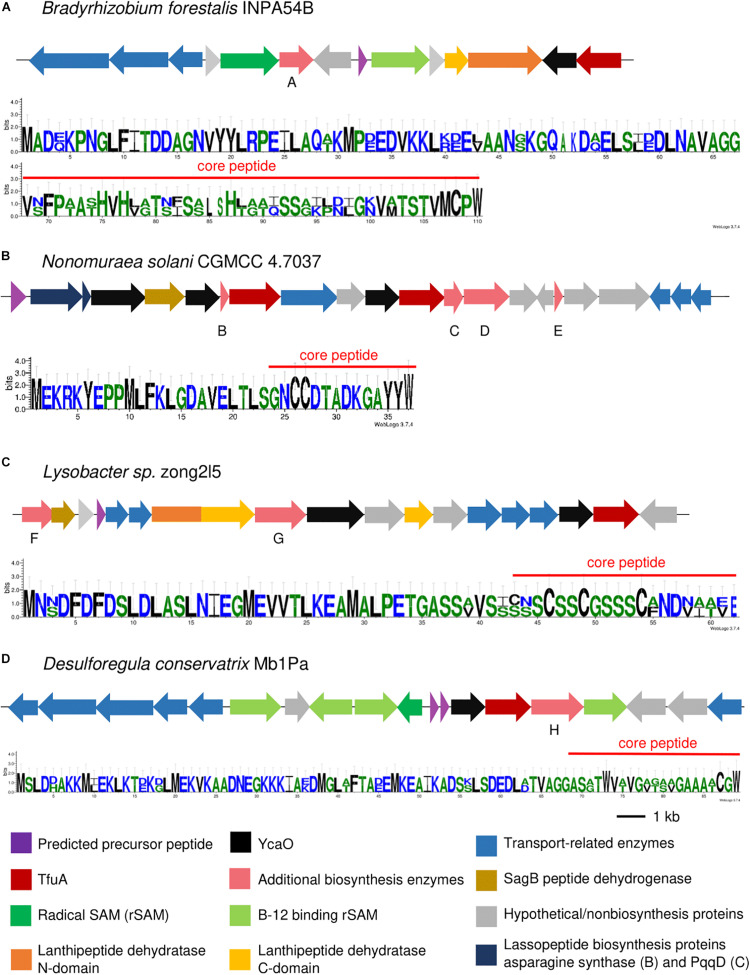
Thioamidated RiPP BGCs containing a precursor peptide annotated as a CCRG-2 family RiPP **(A)**, thiazolylpeptide-type bacteriocin **(B)**, albusnodin family RiPP **(C)**, and Nif11-related precursor **(D)** as predicted by RiPPER. Sequence logo of predicted precursor peptide using RiPPER is indicated. Gene annotations of additional biosynthetic genes are as follows: A: indole prenyltransferase, B: transglutaminase-like superfamily protein, C: thioesterase, D: AMP-binding protein, E: acyl carrier protein, F: molybdopterin-synthase adenylyltransferase MoeB, G: Thiopeptide_F_RRE, and H: glycosyltransferase family 4 protein.

The genome neighborhood was analyzed for each TfuA homolog in the network. The top four GCFs with the largest number of BGCs per phylum exhibit the most common BGC architectures (Figure 4). Species belonging to the genera *Rhizobium, Agrobacterium*, and *Corallococcus* comprise the dominant GCFs detected from Proteobacteria (Supplementary Figures 1–5). Several biosynthesis-related genes, such as glycosyltransferases and ABC transporters, are also common among these GCFs and could be involved in the maturation and transport of the putative peptides encoded by these clusters. GCFs retrieved from Archaea mostly originated from anaerobic methanogens and belong to the genera *Methanosarcina, Methanobrevibacter*, and *Methanothermobacter* (Supplementary Figures 6–10). Most archaeal GCFs contain genes that are implicated in the biosynthesis of other RiPP families, such as radical SAM protein that is involved in the posttranslational modification of RiPPs (Benjdia et al., 2017), and ThiF protein that is required for azoline biosynthesis (Dunbar et al., 2015). *Nostoc* and *Anabaena* primarily constitute clusters from Cyanobacteria (Supplementary Figures 12, 13) and also co-cluster with other RTEs such as bacteriocin biosynthesis proteins. The sequence similarity network of TfuA proteins based on their amino acid sequences was generated by EFI-EST (Supplementary Figure 14). Most of the TfuA proteins are grouped together and show high similarity and conservation among different phyla. However, the TfuA-related BGC architecture shows diversity depending on the phyla and genera.

### Sequence Similarity Network Analysis of Precursor Peptides in TfuA-Related BGCs

Precursor peptides from these unique BGCs were then identified using RiPPER for an accurate depiction of the diversity of putative chemical structures encoded by these BGCs. A total of 7,799 possible precursor peptides were detected, with 5,567 peptides forming 797 clusters and 2,972 singletons after sequence similarity network analysis by using EGN (Halary et al., 2013). This finding indicated a wide variation in the amino acid sequences of the putative precursor peptides (Supplementary Figure 15 and Supplementary Table 5). Consistent with the SNN of BGC sequences, the majority of the peptides are also clustered by taxonomic phylum, which has been observed in the global analysis on the precursor peptides of other RiPP groups (Tietz et al., 2017; Walker et al., 2020). In some large clusters, similarities are observed among precursors originating from different phyla. Thioviridamide-like compounds are clustered together (Santos-Aberturas et al., 2019), although their respective BGC architectures display different gene contents. Alternatively, different peptides can be extracted from BGCs with similar architectures. Despite the TfuA-YcaO pair only targeting MCR, precursor peptides among the identified clusters have been detected in archaeal species (Mahanta et al., 2018).

Nine GCFs were also found to contain putative precursor peptides that share amino acid sequence similarity to other RiPPs of different families. CCRG-2 are secreted small peptides structurally related to the lanthipeptide family prochlorosins. Both CCRG-2 and prochlorosins have only been observed in Cyanobacteria, particularly in *Prochlorococcus* and *Synechococcus* species (Wang et al., 2011; Tang and van der Donk, 2012; Aharonovich and Sher, 2016); however, RiPPER analysis showed that some TfuA-related clusters from *Bradyrhizobium* and *Nostoc* contained putative precursor peptides that show similarity to the CCRG-2 family (Figure 4A and Supplementary Figure 16). The detected precursor peptides also contained the conserved 13 amino acid motif ending with Gly-Gly, which has been found to be involved in the recognition and cleavage of the leader peptide, and export of the mature peptide (Hao Wang et al., 2011; Aharonovich and Sher, 2016). A cluster detected from *Nonomurea solani* contained an albusnodin-like precursor peptide (Figure 4B). Albusnodin, discovered after genome mining of *S. albus*, is the only acetylated lasso peptide reported to date (Zong et al., 2018), although the TfuA-related cluster detected in this study did not contain an acetyltransferase, which is responsible for the acetylation. Precursor peptides that share sequence similarity with characterized thiopeptides were also found in several proteobacterial and actinobacterial species. Detected precursor peptides from several *Rhizobium* and *Herbaspirillum* shared similarity with berninamycin (Supplementary Figure 18), a thiazolyl peptide produced by *Streptomyces bernensis* (Lau and Rinehart, 1994) which displays potent antibacterial activity by disrupting bacterial protein synthesis (Thompson et al., 1982), whereas others were generally annotated as bacteriocins containing thiopeptide-type modifications (Figure 4C and Supplementary Figure 19). Several cyanobacterial and proteobacterial species with genera belonging to *Desulforegula, Anabaena, Rhizobium, Simkania*, and *Ruegeria* contained Nif-11 like precursor peptides in their TfuA-related BGCs (Figure 4D and Supplementary Figures 12, 20), These peptides were named as such as they exhibit similarity from nitrogen fixing proteins from Cyanobacteria. These precursor peptides contained a conserved GG cleavage motif and are found to be associated with lanthionine biosynthesis enzymes (Haft et al., 2010). The BGC from *Desulforegula conservatrix* contained transporters specific to the transport of this family of peptides.

### Several Additional Biosynthetic Genes Associate With Thioamidated RiPPs Biosynthesis

Genome analysis using antiSMASH allows the detection and analysis of possible additional biosynthetic enzymes with their genes close to the core genes and other genes found within the BGC boundary. The identified BGCs contain various tailoring enzymes, including glycosyltransferases, cytochrome P450, oxidoreductases, and hydrolases, a set of enzymes that have not been found on manually annotated thioamide peptide BGCs (Supplementary Figure 21A). The most abundant enzymes are glycosyltransferases. Glycosylated RiPPs are rare, with only a couple of compounds previously reported (Iorio et al., 2014; Wang et al., 2014). Cytochrome P450s are an intriguing enzyme family due to their vast chemical transformations on secondary metabolites (Greule et al., 2018). On RiPPs, P450s are responsible for hydroxylation (Foulston and Bibb, 2010; Zheng et al., 2016), decarboxylation (Crone et al., 2016), epoxidation (Zheng et al., 2016), and cyclopropanation (Gober et al., 2017). SDR family oxidoreductases catalyze to reduce the N-terminal terminal amino acids in several lanthipeptides (Repka et al., 2017). Alpha-beta hydrolases transfer indolyl groups (Qiu et al., 2017) and serve as carboxylesterase (Liao and Liu, 2011) in thiopeptides. These results imply the existence of undiscovered PTMs on these compound classes.

**FIGURE 5:**
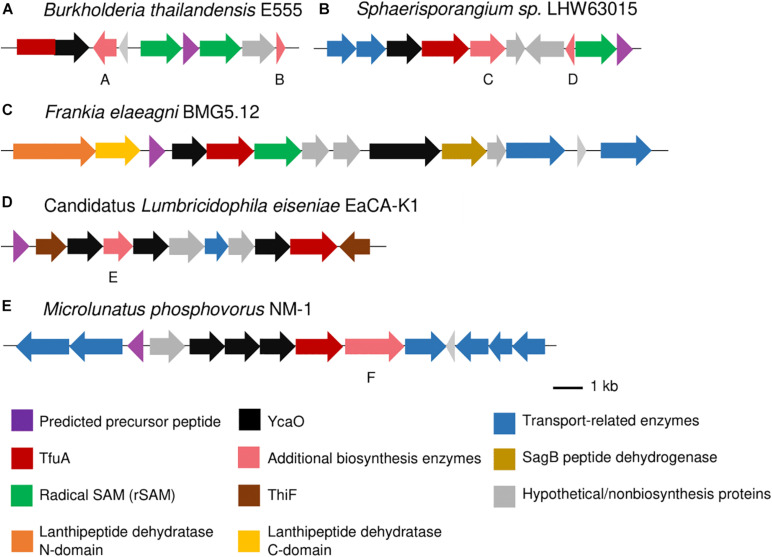
Thioamidated RiPP BGCs containing a fused *tfuA-ycaO* gene **(A)**, annotated to produce ranthipeptides **(B)**, linear azole containing peptides (LAP) **(C)**, thiopeptides **(D)**, and with multiple uncharacterized *ycaO* genes **(E)** as annotated by antiSMASH. Sequence logo of predicted precursor peptides are found in the Supplementary Information. Gene annotations are as follows: A: phytanoyl-CoA dioxygenase family protein, B: ThiS, C: cytochrome P450, D: PqqD family protein (Stand_Alone_Lasso_RRE PF05402), E: nitroreductase (PF00881), and F: prolyl endopeptidase (Peptidase S9).

RiPP-specific additional biosynthetic enzymes that could lead to the installation of other posttranslational modifications on the putative thioamidated peptides were also found in the BGCs, especially in Proteobacteria where more diverse BGC architectures were observed. Several *Sinorhizobium* species contained a gene encoding for a heme oxygenase-like protein (Supplementary Figure 22), similar to that observed in the thiovarsolin BGC (Santos-Aberturas et al., 2019), which is responsible for the dehydrogenation of thiovarsolins. Fused *tfuA-ycaO* genes were also detected in *Burkholderia thailandensis* alongside two RiPP-specific radical S-adenosyl-L-methionine (rSAM) proteins that could be involved in the biosynthesis of the peptide (Figure 5A and Supplementary Figure 23), although a specific function cannot be assigned to these rSAM proteins as they do not share similarity to any characterized protein. Radical SAM proteins have been implicated in imparting diverse PTMs on RiPPs (Benjdia et al., 2017), and thus could take part in the further modification of thioamidated peptides. Two GCFs from *Sphaerisporangium, Microbispora* and *Herbidospora* each contained a rSAM protein that was further annotated by antiSMASH to produce ranthipeptides based from the presence of a SPASM domain in the rSAM protein and a standalone PqqD protein (Figure 5B and Supplementary Figure 24). PqqD is a RiPP precursor peptide Recognition Element (RRE), although functionally characterized rSAM enzymes that generate thioether bond formation show that PqqD should exist as an N-terminal domain of the rSAM protein rather than a standalone protein (Mahanta et al., 2017). Other putative additional biosynthetic enzymes cytochrome P450 and O-methyltransferase were also found in both of these GCFs, and the predicted precursor peptide contained several Cys and Ser residues that can participate in the installation of the thioether linkages (Hudson et al., 2019). GCFs from *Bradyrhizobium* and *Desulforegula* species contained rSAM proteins containing a B_12_-binding domain (Figures 4A,D and Supplementary Figure 16), which denotes a possible methylation on the produced RiPP (Parent et al., 2016; Mahanta et al., 2017).

In addition to the *ycaO* gene adjacent to the *tfuA* gene, several BGCs have additional *ycaO* genes that can further install modifications on the putative thioamidated peptide. RiPP BGCs containing a cyclodehydratase usually encoded in part by a *ycaO* gene and a flavin-dependent dehydrogenase can possibly lead to the production of linear azole-containing peptides (Burkhart et al., 2015, 2017, p.). These elements were found in some BGCs detected in this study (Figure 5C and Supplementary Figures 19, 25), most of which contained a fused *ycaO* and cyclodehydratase domains, and split lanthipeptide dehydratases that could catalyze the dehydration of serine and threonine residues on the RiPP, as observed in goadsporin biosynthesis (Ozaki et al., 2016; Burkhart et al., 2017). On the other hand, thiopeptide biosynthesis requires the presence of a ThiF-like protein, which serves as the RRE that binds the precursor peptide, split lanthipeptide dehydratases, and an enzyme that can perform a (4 + 2) cycloaddition for the formation of the macrocycle (Burkhart et al., 2017). Thiopeptides that contain thioamides catalyzed by TfuA-YcaO include saalfelduracin, thiopeptin, and Sch 18640 (Schwalen et al., 2018). BGCs encoding for putative thiopeptides were also detected from the clusters identified in this study, mostly having an extra C-terminal lanthipeptide dehydratase domain as the cycloaddition enzyme (Figures 4C, 5D and Supplementary Figure 26). Some BGCs with multiple *ycaO* genes lacked other additional biosynthetic enzymes and specific domains to properly predict the reaction they could catalyze (Figure 5E and Supplementary Figure 27). Clusters containing two *tfuA* genes were also observed, with a cluster from *Nonomurea solani* harboring a second *tfuA* gene with a protein-L-isoaspartate (D-aspartate) O-methyltransferase (PCMT) domain (Figure 4B and Supplementary Figure 17) and *Streptacidiphilus carbonis* NBRC 100919 with two *tfuA* genes and three *ycaO* genes (Supplementary Figure 27).

The frequency of other genes found in the BGCs prompted the analysis for other common co-occurring enzymatic activities that might be involved in peptide biosynthesis (Supplementary Figure 21B). Several transcriptional regulators and transporters can be found in the cluster that might be responsible for the regulation and export of the compound, respectively. ABC transporters are one of the main resistance mechanisms of bacteria from self-toxicity from the produced RiPPs. This process is performed through the combined cleavage of the inactive leader peptide and their export, such as ATP-binding ABC transporters or transport of the mature peptide itself (Arnison et al., 2013). Although an MFS transporter gene can be found in thiovarsolin BGC, deletion experiments have not disrupted compound production (Santos-Aberturas et al., 2019). The absence of any transport-related proteins from the BGCs of known thioamidated peptides suggests that transporters suggests that specific transporters might not be required for export of some classes of thioamidated RiPPs.

### Phylogenetic Analysis Reveals the Horizontal Gene Transfer of *tfuA*

**FIGURE 6:**
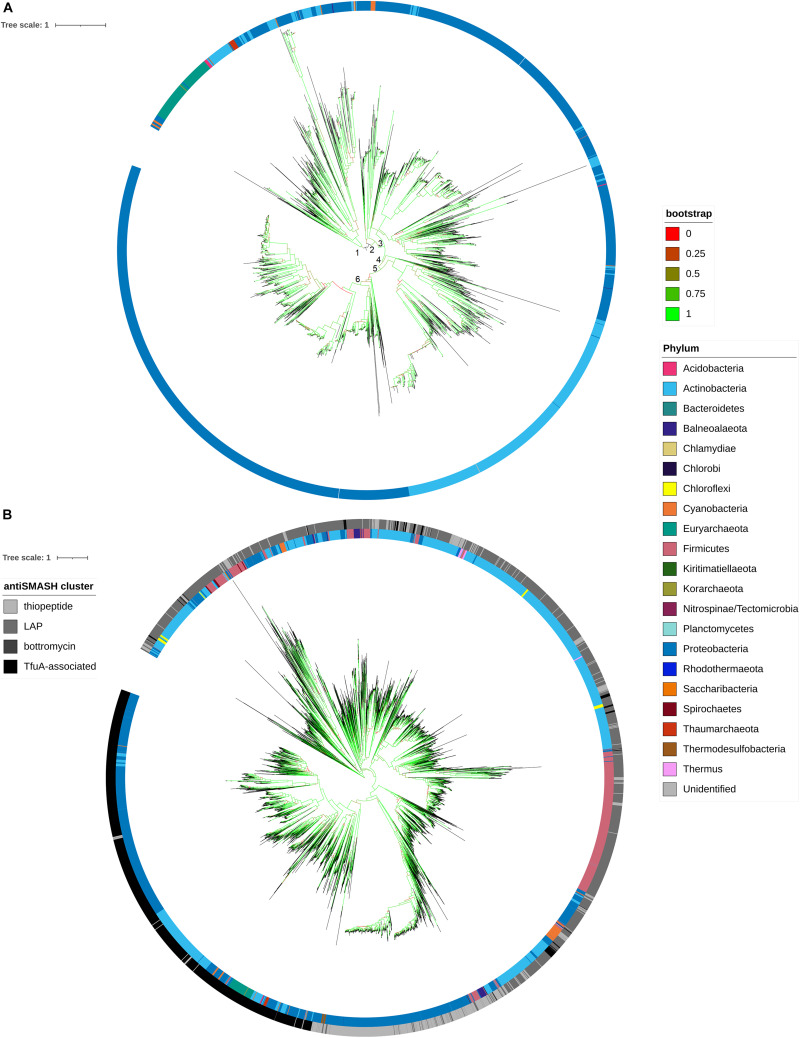
Molecular phylogenetic distribution of filtered TfuA protein sequences from TfuA-related BGCs **(A)** and filtered YcaO protein sequences from YcaO-containing BGCs (thiopeptides, linear azoline containing peptides (LAP), bottromycins, and TfuA-associated) **(B)** using a midpoint rooted approximately maximum likelihood phylogenetic analysis.

Phylogenetic relationships among all the detected BGCs were identified from the sequence comparison of protein sequences of TfuA. The established robust phylogenetic tree shows that TfuA diverges into two clades. Clade 1 contains most of the bacterial and archaeal phyla, while clade 2 comprises mostly sequences retrieved from Proteobacteria and Actinobacteria (Figure 6A). As suggested by the scattering of sequences coming from different phyla, clade 1 indicates horizontal gene transfer between its members. This phenomenon can also be observed from the clustering of several BGCs from different phyla. Several subgroups (groups 3–6) are derived from clade 2. Group 5 represents actinobacterial strains, whereas groups 3, 4, and 6 contain sequences mostly from BGCs identified from proteobacterial species. Known TfuA sequences that produce thioamidated peptides belong to clade 1. Diversification of these gene clusters is possibly driven by recombination, gene duplication, gene deletion, and subsequent mutation, followed by natural selection. Thus, further experimental validation is proposed for the members of the other clade to determine whether this divergence has led to a drastic change in enzyme function. A phylogenetic tree of protein sequences of YcaO from thioamidated RiPP BGCs and other antiSMASH BGCs containing YcaO was constructed (Figure 6B). The topology showed division of sequences into clades according to the predicted RiPP they putatively produce. This is due to the presence of specific protein domains in the amidine or azoline forming YcaO proteins that perform heterocyclyzation. It is important to note that antiSMASH usually annotates thiopeptide-encoding BGCs and cyanobactins as LAP BGCs due to the similarity of the core proteins used for their biosynthesis. Nonetheless, a clade composed of Tfu-associated YcaO proteins is clearly defined. The distribution of phyla within this Tfu-associated YcaO protein clade also shows a similar topology as to that of in the phylogenetic analysis of TfuA proteins, which suggests that these two proteins are strongly associated.

### Protein Sequence Motifs Are Enriched in TfuA-Associated YcaO

**FIGURE 7:**
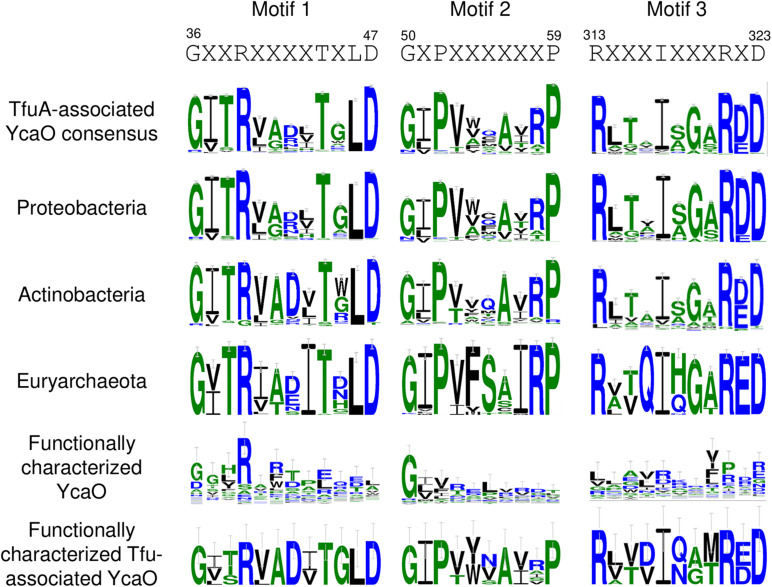
Sequence logo of specific protein sequence motifs identified from TfuA-associated YcaO proteins.

To distinguish YcaO proteins that participate in thioamide formation to those that give rise to azole or azoline biosynthesis, 2,422 *tfuA*-associated *ycaO* genes were extracted from the gathered BGCs, translated into protein sequences, and were analyzed using MEME (Bailey et al., 2009) to identify specific conserved protein sequence motifs that are absent in non-*tfuA* associated *ycaO* genes. Together with the previously described three ATP-binding motifs found in all YcaO proteins (Dunbar et al., 2014), three motifs were identified that were not found on other *ycaO* genes by comparison with the multiple sequence alignments of 50 functionally characterized non-*tfuA* associated *ycaO* genes extracted from MiBIG (Kautsar et al., 2020) and from the 20 member proteins used in constructing the COG domain model for YcaO (Lu et al., 2020; Figure 7). Motifs 1 and 2 are located upstream of the first ATP-binding motif, whereas motif 3 is placed five residues after the last ATP-binding motif. Comparison with the resolved crystal structure of a YcaO enzyme responsible for thioamidation of MCR (Dong et al., 2019) showed that motifs 1 and 2 participate in the formation of both the third α-helix and third β-sheet respectively, while motif 3 is involved in the formation of another β-sheet together with the third ATP-binding motif. Although these motifs do not contain catalytic residues, their conservation among different phyla and absence on non-*tfuA* associated *ycaO* genes suggests that these motifs are an important feature of TfuA-associated YcaO proteins. Comparison of the ATP-binding motifs on the other hand showed several preferred amino acids, such as the Met-84 residue in motif 1, His-188 in motif 2, and Ala-305 in motif 3 (Supplementary Figure 28).

## Conclusion

The immense diversity in the thioamidated RiPP biosynthesis gene clusters in different phyla has been highlighted through global genome mining. The widespread co-occurrence of TfuA and YcaO proteins in diverse microorganisms reveals the presence of such thioamidated secondary metabolite biosynthetic pathways in various bacterial and archaeal phyla. This work is the first to report the presence of unique thioamidated RiPP biosynthesis gene clusters belonging to phyla other than Actinobacteria, most of which originate from phylum Proteobacteria. Several BGCs which could putatively produce highly modified thioamidated RiPPs were identified. Protein sequence motifs were also identified from *ycaO* genes that are associated with *tfuA* genes as compared to *ycaO* genes implicated in amidine or azoline biosynthesis. These results have further expanded the rich diversity of thioamidated RiPP biosynthesis gene clusters which should be subjected for further study.

## Data Availability Statement

Genome data was downloaded from NCBI Assembly. Accession numbers can be found in Supplementary Table 1.

## Author Contributions

JJLM and P-YQ designed the study. JJLM and CW performed all the experiments. JJLM and L-LL analyzed the data and drafted the manuscript. L-LL reviewed and edited the manuscript. All authors have read and agreed to the published version of the manuscript.

## Conflict of Interest

The authors declare that the research was conducted in the absence of any commercial or financial relationships that could be construed as a potential conflict of interest.
