# Supplementary material for: Global Genome Mining Reveals the Distribution of Diverse Thioamidated RiPP Biosynthesis Gene Clusters
Source: Front Microbiol. 2021 Apr 30;12:635389. doi: 10.3389/fmicb.2021.635389 (PMC8120280; doi:10.3389/fmicb.2021.635389)

Supplementary Material

Global genome mining reveals the distribution of diverse thioamidated RiPP biosynthetic gene clusters

Jessie James Limlingan Malit[1], Chuanhai Wu[1], Ling-Li Liu[1, 2]*, Pei-Yuan Qian[1,]*

[1] Department of Ocean Science, and Hong Kong Branch of Southern Marine Science and Engineering Guangdong Laboratory, The Hong Kong University of Science and Technology, Hong Kong, China

[2] Shaanxi Key Laboratory of Natural Products & Chemical Biology, College of Chemistry & Pharmacy, Northwest A&F University, 22 Xinong Road, Yangling 712100, Shaanxi, People’s Republic of China

*Correspondence: boqianpy@ust.hk; leonie@nwsuaf.edu.cn; Tel.: +852-23587331

Index

Figure S1. Conserved gene cluster architecture of TfuA - YcaO gene cluster families with most members in Proteobacteria *Rhizobium sp*. Genes in bold indicate additional biosynthetic or transport genes as annotated by antiSMASH. Sequence logos of predicted precursor peptides using RiPPER are indicated. 5

Figure S2. Conserved gene cluster architecture of TfuA - YcaO gene cluster families with most members in Proteobacteria, *Rhizobium sp*. Genes in bold indicate additional biosynthetic or transport genes as annotated by antiSMASH. Sequence logos of predicted precursor peptides using RiPPER are indicated. 6

Figure S3. Conserved gene cluster architecture of TfuA - YcaO gene cluster families with most members in Proteobacteria, *Agrobacterium sp.* Genes in bold indicate additional biosynthetic or transport genes as annotated by antiSMASH. Sequence logos of predicted precursor peptides using RiPPER are indicated. 7

Figure S4. Conserved gene cluster architecture of TfuA - YcaO gene cluster families with most members in Proteobacteria, *Rhizobium sp.* Genes in bold indicate additional biosynthetic or transport genes as annotated by antiSMASH. Sequence logos of predicted precursor peptides using RiPPER are indicated. 8

Figure S5. Conserved gene cluster architecture of TfuA - YcaO gene cluster families with most members in Proteobacteria, *Corallococcus sp.* Genes in bold indicate additional biosynthetic or transport genes as annotated by antiSMASH. Sequence logos of predicted precursor peptides using RiPPER are indicated. 9

Figure S6. Conserved gene cluster architecture of TfuA - YcaO gene cluster families with most members in Euryarchaeota, *Methanosarcina sp.* Genes in bold indicate additional biosynthetic or transport genes as annotated by antiSMASH. 10

Figure S7. Conserved gene cluster architecture of TfuA - YcaO gene cluster families with most members in Euryarchaeota, *Methanobrevibacter sp.* Genes in bold indicate additional biosynthetic or transport genes as annotated by antiSMASH. 11

Figure S8. Conserved gene cluster architecture of TfuA - YcaO gene cluster families with most members in Euryarchaeota, *Methanothermobacter sp.* Genes in bold indicate additional biosynthetic or transport genes as annotated by antiSMASH. 12

Figure S9. Conserved gene cluster architecture of TfuA - YcaO gene cluster families with most members in Euryarchaeota, *Methanolobus sp.* Genes in bold indicate additional biosynthetic or transport genes as annotated by antiSMASH. 13

Figure S10. Conserved gene cluster architecture of TfuA - YcaO gene cluster families with most members in Euryarchaeota, *Methanoculleus sp.* Genes in bold indicate additional biosynthetic or transport genes as annotated by antiSMASH. 14

Figure S11. Conserved gene cluster architecture of TfuA - YcaO gene cluster families with most members in Thaumarchaeota *Nitrosocosmicus sp.*. Genes in bold indicate additional biosynthetic or transport genes as annotated by antiSMASH. 15

Figure S12. Conserved gene cluster architecture of TfuA - YcaO gene cluster families with most members in Cyanobacteria. Genes in bold indicate additional biosynthetic or transport genes as annotated by antiSMASH. Sequence logos of predicted precursor peptides using RiPPER are indicated. 16

Figure S13. Conserved gene cluster architecture of TfuA - YcaO gene cluster families with most members in Cyanbobacteria *Nostoc sp.*. Genes in bold indicate additional biosynthetic or transport genes as annotated by antiSMASH. Sequence logos of predicted precursor peptides using RiPPER are indicated. 17

Figure S14. Sequence similarity network of TfuA protein sequences generated using EFI-EST. 18

Figure S15. Sequence similarity network of precursor peptides identified through RiPPER within TfuA-related BGCs. 19

Figure S16. TfuA-related GCFs containing an CCRG-2 family-like precursor peptide as predicted by RiPPER. GCF from *Nostoc sp.* share the same predicted precursor peptide network. Sequence logo of predicted precursor peptide using RiPPER is indicated. 20

Figure S16 (cont). TfuA-related GCFs containing an CCRG-2 family-like precursor peptide as predicted by RiPPER. GCFs from *Nostoc* share the same predicted precursor peptide network. Sequence logo of predicted precursor peptide using RiPPER is indicated. 21

Figure S17. TfuA-related GCFs containing an albusnodin-like precursor peptide as predicted by RiPPER. Sequence logo of predicted precursor peptide using RiPPER is indicated. 22

Figure S18. TfuA-related GCFs containing berninamycin-like precursor peptide as predicted by RiPPER. GCFs from *Rhizobium* share the same predicted precursor peptide network. Sequence logo of predicted precursor peptide using RiPPER is indicated. 23

Figure S19. TfuA-related GCF containing with the precursor peptide annotated as a bacteriocin containing thiopeptide-type modifications as predicted by RiPPER. Sequence logo of predicted precursor peptide using RiPPER is indicated. 24

Figure S20. TfuA-related GCFs containing Nif-like precursor peptides as predicted by RiPPER. 25

Figure S21. Frequencies of additional modification genes (A) and other genes (B) as annotated by antiSMASH found within TfuA-related BGCs. 26

Figure S22. TfuA-related GCF containing an iron-containing redox enzyme family protein. Sequence logo of predicted precursor peptide using RiPPER is indicated. 27

Figure S23. TfuA-related GCF containing a fused *tfuA* and *ycaO* genes. Sequence logo of predicted precursor peptide using RiPPER is indicated. 28

Figure S24. TfuA-related GCF annotated to also produce ranthipeptides as annotated by antiSMASH. Both GCFs share the same precursor peptide network. Sequence logo of predicted precursor peptide using RiPPER is indicated. 29

Figure S25. TfuA-related GCF annotated to also produce linear azole-containing peptides as annotated by antiSMASH. Sequence logo of predicted precursor peptide using RiPPER is indicated. 30

Figure S26. TfuA-related GCFs annotated to also produce thiopeptides as annotated by antiSMASH. Sequence logo of predicted precursor peptide using RiPPER is indicated. 31

Figure S26 (cont.). TfuA-related GCFs annotated to also produce thiopeptides as annotated by antiSMASH. Sequence logo of predicted precursor peptide using RiPPER is indicated. 32

Figure S27. TfuA-related GCFs containing multiple *ycaO* genes without further annotation. Sequence logo of predicted precursor peptide using RiPPER is indicated. 33

Figure S27 (cont.). TfuA-related GCFs containing multiple *ycaO* genes without further annotation. Sequence logo of predicted precursor peptide using RiPPER is indicated. 34

Figure S28. Sequence logo of the ATP-binding sequence motifs identified from TfuA-associated YcaO proteins. 35

Figure S1. Conserved gene cluster architecture of TfuA - YcaO gene cluster families with most members in Proteobacteria *Rhizobium sp.* Genes in bold indicate additional biosynthetic or transport genes as annotated by antiSMASH. Sequence logos of predicted precursor peptides using RiPPER are indicated.

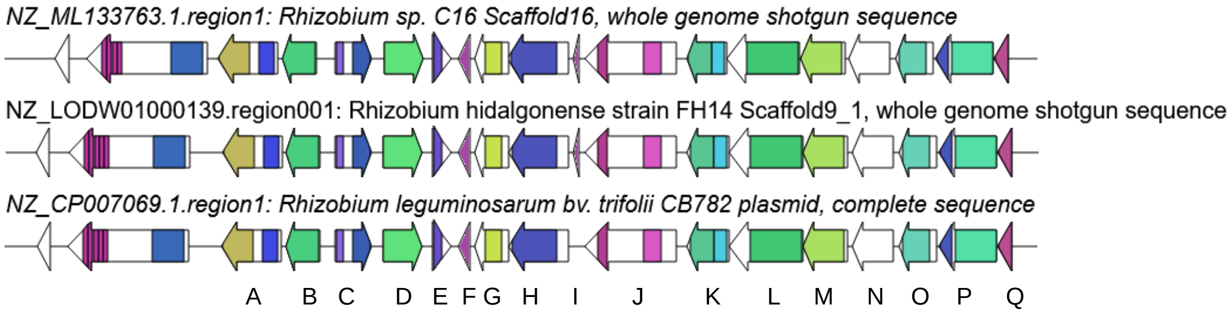

A: L-fuconate dehydratase
B: SDR family oxidoreductase
C: IclR family transcriptional regulator
D: amidohydrolase
E: helix-turn-helix transcriptional regulator
F: putative precursor peptide 1
G: TfuA
H: YcaO
I: putative precursor peptide 2
J: tetratricopeptide repeat protein
K: carboxylating nicotinate-nucleotide diphosphorylase
L: L-aspartate oxidase
M: quinolinate synthase NadA
N: hypothetical protein
O: 3-methyl-2-oxobutanoate hydroxymethyltransferase
P: FAD-dependent oxidoreductase
Q: Rieske 2Fe-2S domain-containing protein

Putative precursor peptide 1 (Cluster 5)

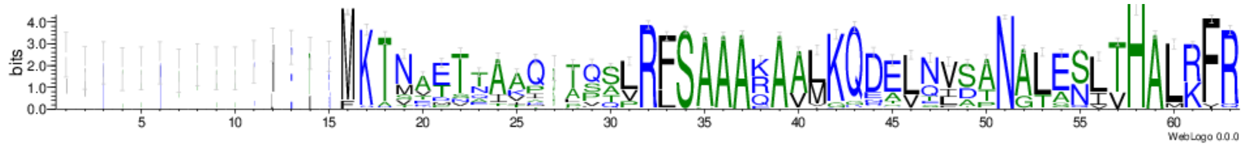

Putative precursor peptide 2 (Cluster 6)

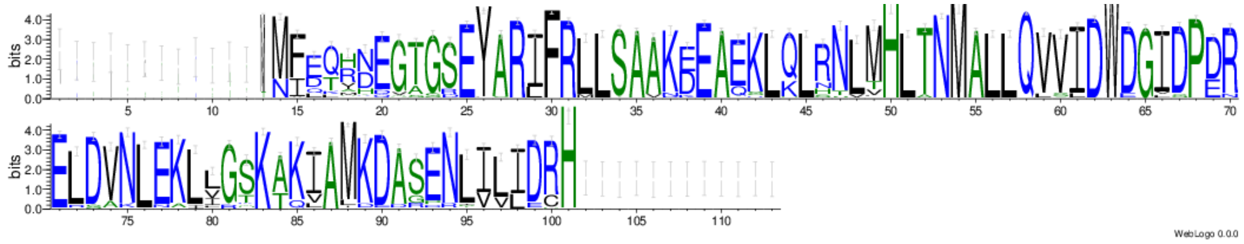

Figure S2. Conserved gene cluster architecture of TfuA - YcaO gene cluster families with most members in Proteobacteria, *Rhizobium sp*. Genes in bold indicate additional biosynthetic or transport genes as annotated by antiSMASH. Sequence logos of predicted precursor peptides using RiPPER are indicated.

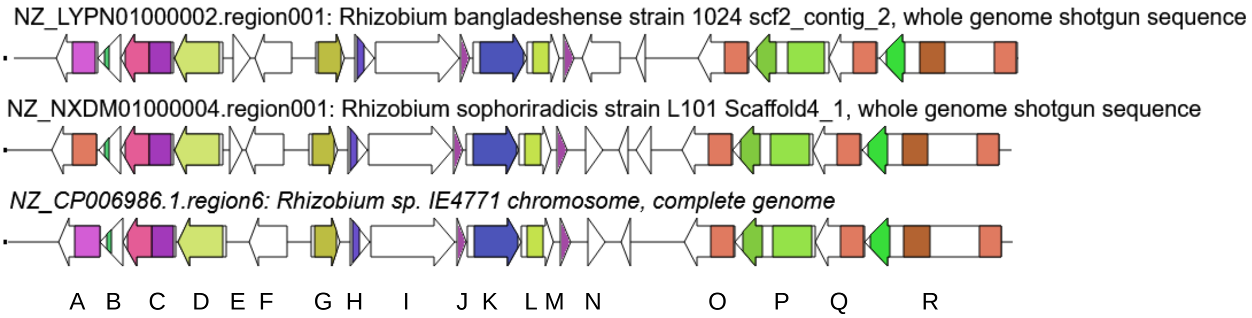

A: polysaccharide pyruvyl transferase family protein
B: acyltransferase
C: glycosyltransferase
D: endo-1,4-beta-xylanase
E: hypothetical protein
F: exopolysaccharide biosynthesis protein
G: class I SAM-dependent methyltransferase
H: helix-turn-helix domain-containing protein
I: peptide antibiotic resistance protein
J: putative precursor peptide 1
K: YcaO
L: TfuA
M: putative precursor peptide 2
N: hypothetical protein
O: glycosyltransferase family 2 protein
P: ABC transporter ATP-binding protein
Q: glycosyltransferase family 2 protein
R: glycosyltransferase

Putative precursor peptide 1 (Cluster 1)

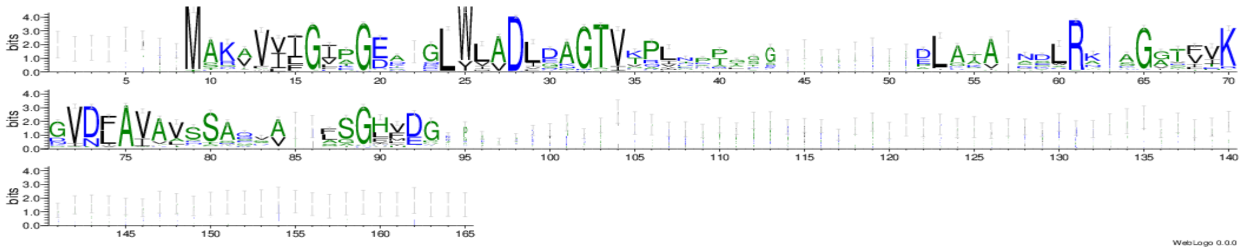

Putative precursor peptide 2 (Cluster 4)

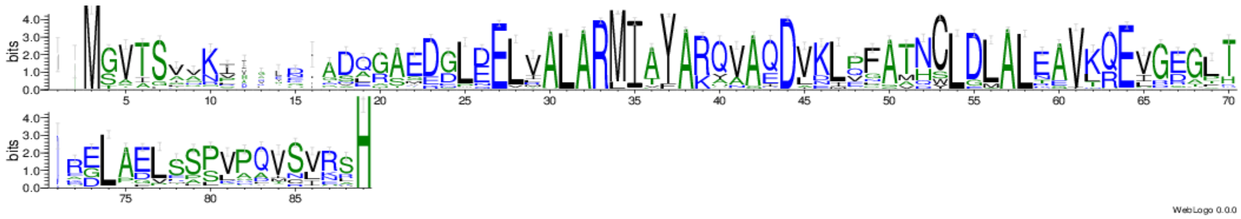

Figure S3. Conserved gene cluster architecture of TfuA - YcaO gene cluster families with most members in Proteobacteria, *Agrobacterium sp.* Genes in bold indicate additional biosynthetic or transport genes as annotated by antiSMASH. Sequence logos of predicted precursor peptides using RiPPER are indicated.

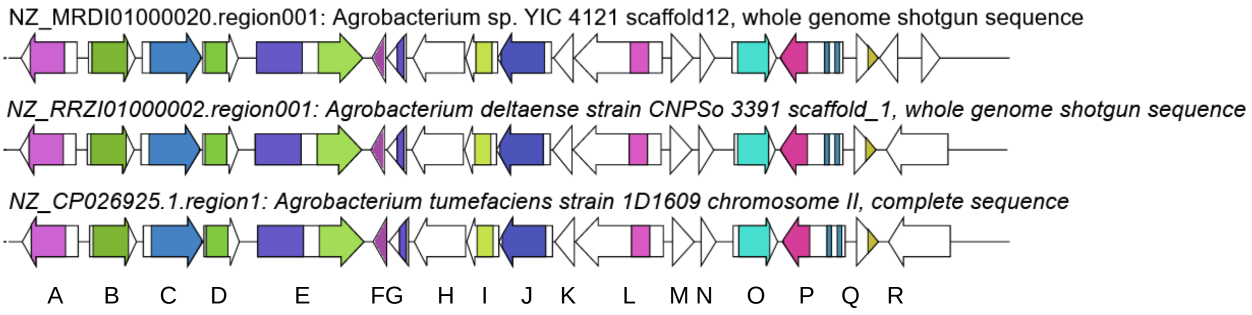

A: ROK family protein
B: D-xylose ABC transporter substrate-binding protein
C: sugar ABC transporter permease
D: sugar ABC transporter ATP-binding protein
E: 5-methyltetrahydropteroyltriglutamate--homocysteine S-methyltransferase
F: putative precursor peptide
G: helix-turn-helix transcriptional regulator
H: hypothetical protein
I: TfuA
J: YcaO
K: hypothetical protein
L: SARP family transcriptional regulator
M: hypothetical protein
N: hypothetical protein
O: TIM barrel protein
P: family 16 glycosylhydrolase
Q: transcription elongation factor GreA
R: SGNH/GDSL hydrolase family protein

Putative precursor peptide 2 (Cluster 3)

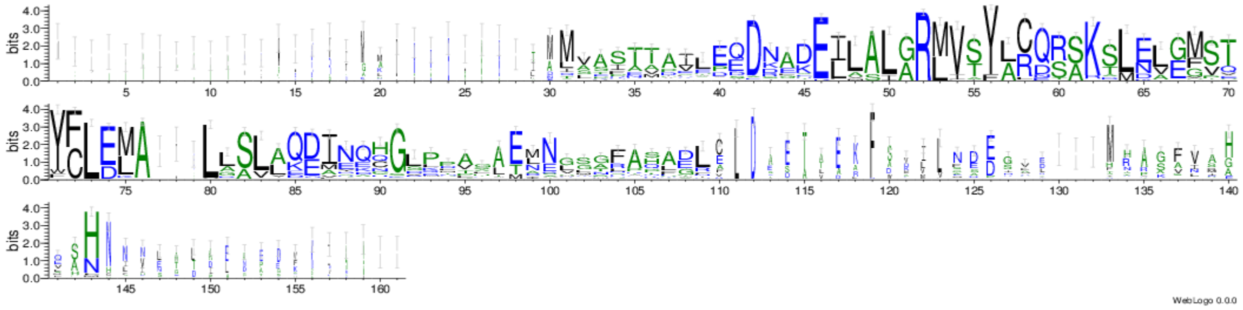

Figure S4. Conserved gene cluster architecture of TfuA - YcaO gene cluster families with most members in Proteobacteria, *Rhizobium sp.* Genes in bold indicate additional biosynthetic or transport genes as annotated by antiSMASH. Sequence logos of predicted precursor peptides using RiPPER are indicated.

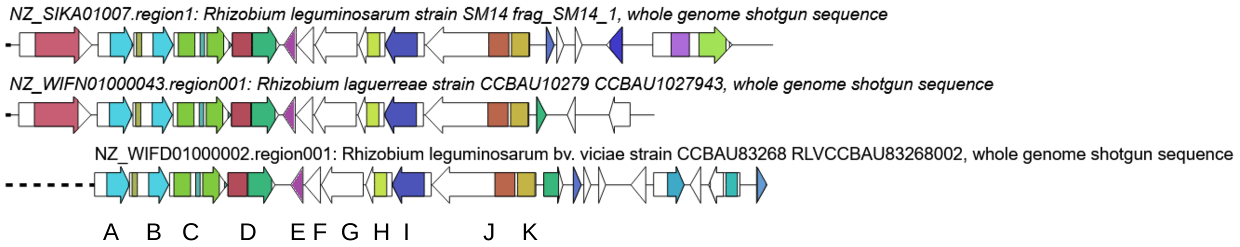

A: **ABC transporter ATP-binding protein**
B: **ABC transporter permease**
C: ABC transporter ATP-binding protein
D: alpha-glucosidase/alpha-galactosidase
E: precursor peptide
F: hypothetical protein
G: hypothetical protein
H: **TfuA**
I: YcaO
J: AAA family ATPase
K: alpha-glucosidase/alpha-galactosidase

Putative precursor peptide (Cluster 23)

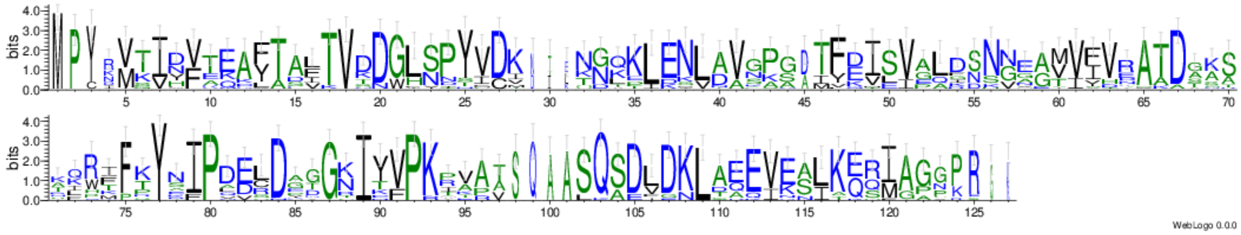

Figure S5. Conserved gene cluster architecture of TfuA - YcaO gene cluster families with most members in Proteobacteria, *Corallococcus sp*. Genes in bold indicate additional biosynthetic or transport genes as annotated by antiSMASH. Sequence logos of predicted precursor peptides using RiPPER are indicated.

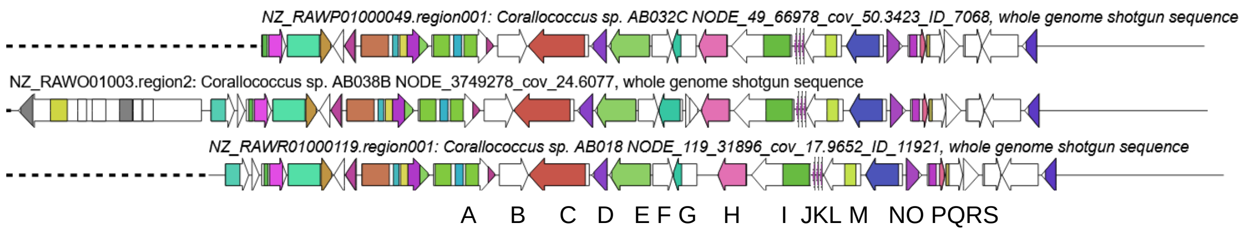

A: ABC-F family ATP-binding cassette domain-containing protein
B: flagellar motor protein MotB
C: OPT/YSL family transporter
D: peptidylprolyl isomerase
E: dicarboxylate/amino acid:cation symporter
F: hypothetical protein
G: site-specific DNA-methyltransferase
H: DUF2381 family protein
I: serine/threonine protein kinase
J: putative precursor peptide 1
K: putative precursor peptide 2
L: putative precursor peptide 3
M: TfuA
N: YcaO
O: peroxiredoxin
P: sigma-70 family RNA polymerase sigma factor
Q: zf-HC2 domain-containing protein
R: hypothetical protein
S: DUF4136 domain-containing protein

Putative precursor peptide 1 (Cluster 100)

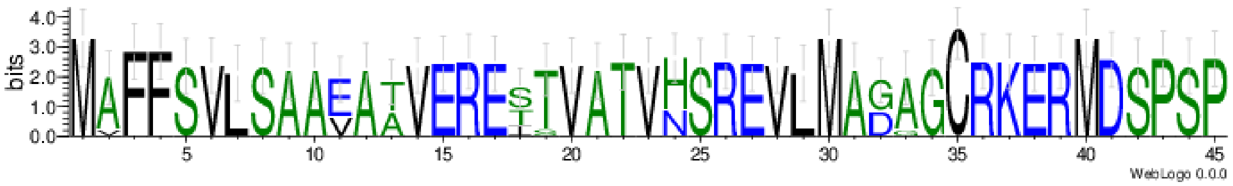

Putative precursor peptide 2 (Cluster 218)

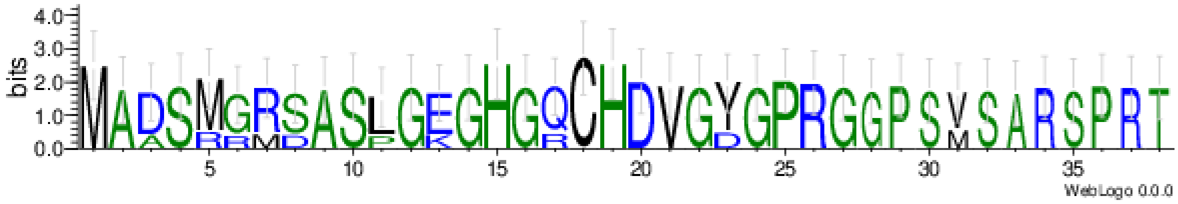

Putative precursor peptide 3 (Cluster 612)

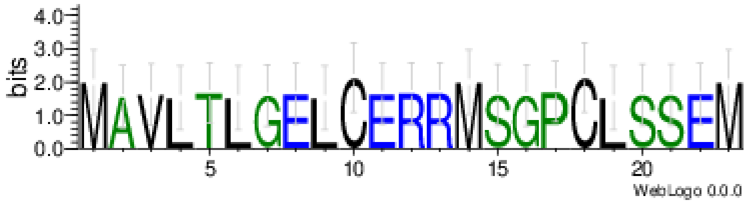

Figure S6. Conserved gene cluster architecture of TfuA - YcaO gene cluster families with most members in Euryarchaeota, *Methanosarcina sp.* Genes in bold indicate additional biosynthetic or transport genes as annotated by antiSMASH.

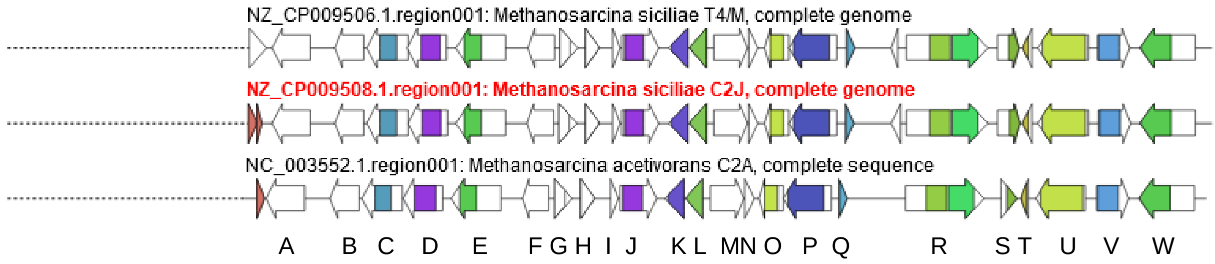

A: polysaccharide deacetylase family protein
B: 3-methylornithyl-N6-L-lysine dehydrogenase PylD
C: 3-methylornithine--L-lysine ligase PylC
D: methylornithine synthase PylB
E: pyrrolysine--tRNA(Pyl) ligase
F: hypothetical protein
G: DUF134 domain-containing protein
H: DUF5320 domain-containing protein
I: hypothetical protein
J: radical SAM protein
K: ferritin
L: universal stress protein
M: hypothetical protein
N: hypothetical protein
O: TfuA
P: YcaO
Q: DUF2892 domain-containing protein
R hypothetical protein
S: copper-translocating P-type ATPase
T: cytochrome c
U: PLDc N-terminal domain-containing protein
V: MFS transporter
W: class I SAM-dependent methyltransferase

Figure S7. Conserved gene cluster architecture of TfuA - YcaO gene cluster families with most members in Euryarchaeota, *Methanobrevibacter sp*. Genes in bold indicate additional biosynthetic or transport genes as annotated by antiSMASH.

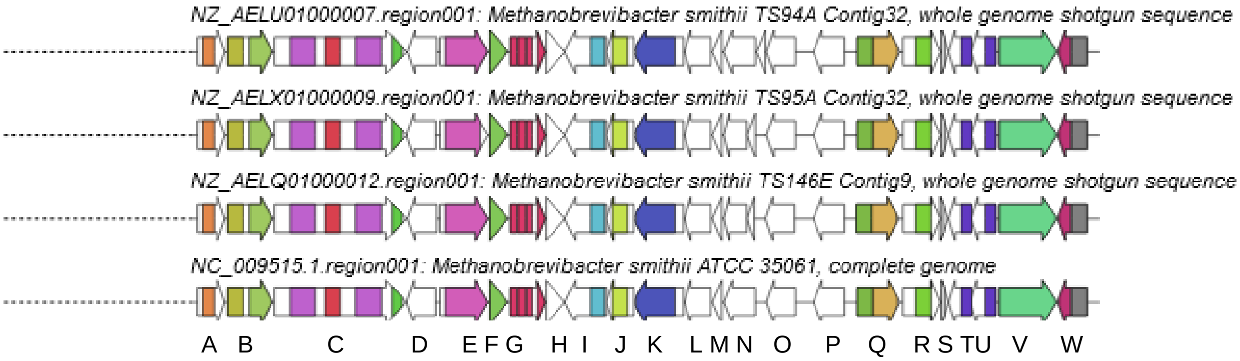

A: class I SAM-dependent methyltransferase
B: glutamine-hydrolyzing carbamoyl-phosphate synthase small subunit
C: carbamoyl-phosphate synthase large subunit
D: zinc ribbon domain-containing protein
E: amidohydrolase family protein
F: universal stress protein
G: CBS domain-containing protein
H: hypothetical protein
I: ATP-dependent sacrificial sulfur transferase LarE
J: TfuA
K: YcaO

L: site-2 protease family protein
M: hypothetical protein
N: hypothetical protein
O: hypothetical protein
P: hypothetical protein
Q: aminopeptidase P family protein
R: type II secretion system F family protein
S: class III signal peptide-containing protein
T: DUF1211 domain-containing protein
U: DUF1211 domain-containing protein
V: aldehyde dehydrogenase family protein
W: tRNA-binding protein

Figure S8. Conserved gene cluster architecture of TfuA - YcaO gene cluster families with most members in Euryarchaeota, *Methanothermobacter sp.* Genes in bold indicate additional biosynthetic or transport genes as annotated by antiSMASH.

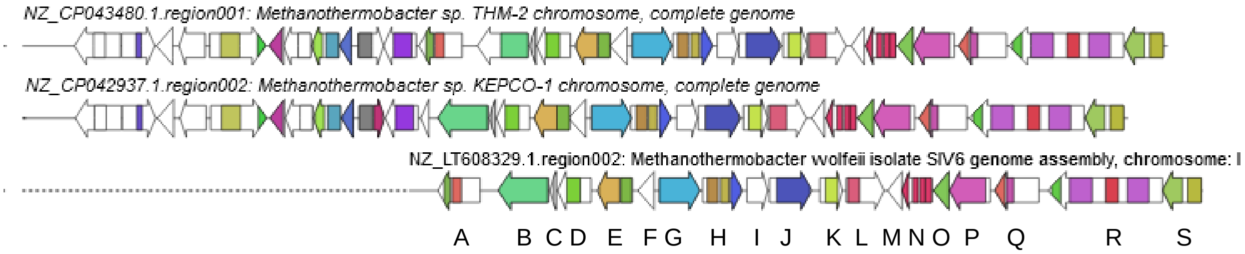

A: chitobiase/beta-hexosaminidase C-terminal domain-containing protein
B: aldehyde dehydrogenase family protein
C: class III signal peptide-containing protein
D: type II secretion system F family protein
E: aminopeptidase P family protein
F: HIT family protein
G: iron-containing alcohol dehydrogenase
H: PAS domain S-box protein
I: site-2 protease family protein
J: YcaO
K: TfuA
L: ATP-dependent sacrificial sulfur transferase LarE
M: hypothetical protein
N: CBS domain-containing protein
O: universal stress protein
P: amidohydrolase family protein
Q: PKD domain-containing protein
R: carbamoyl-phosphate synthase large subunit
S: glutamine-hydrolyzing carbamoyl-phosphate synthase small subunit

Figure S9. Conserved gene cluster architecture of TfuA - YcaO gene cluster families with most members in Euryarchaeota, *Methanolobus sp*. Genes in bold indicate additional biosynthetic or transport genes as annotated by antiSMASH.

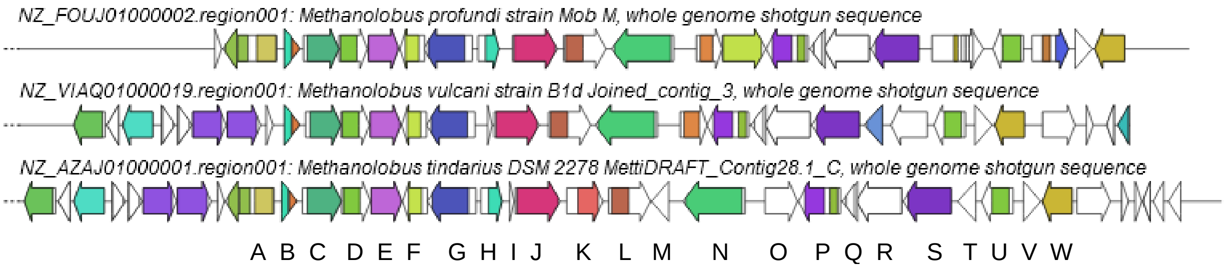

A: MBL fold metallo-hydrolase
B: metal-dependent transcriptional regulator
C: zinc ABC transporter substrate-binding protein
D: ABC transporter ATP-binding protein
E: metal ABC transporter permease
F: TfuA
G: YcaO
H: cytochrome P460 family protein
I: hypothetical protein
J: cysteine desulfurase
K: IS5 family transposase
L: ORC1-type DNA replication protein
M: hypothetical protein
N: thermosome subunit
O: YkgJ family cysteine cluster protein
P: TIGR04013 family B12-binding domain/radical SAM domain-containing protein
Q: DUF126 domain-containing protein
R: DUF521 domain-containing protein
S: UbiD family decarboxylase
T: hypothetical protein
U: ATP-binding cassette domain-containing protein
V: hypothetical protein
W: 4,5-DOPA dioxygenase extradiol

Figure S10. Conserved gene cluster architecture of TfuA - YcaO gene cluster families with most members in Euryarchaeota, *Methanoculleus sp*. Genes in bold indicate additional biosynthetic or transport genes as annotated by antiSMASH.

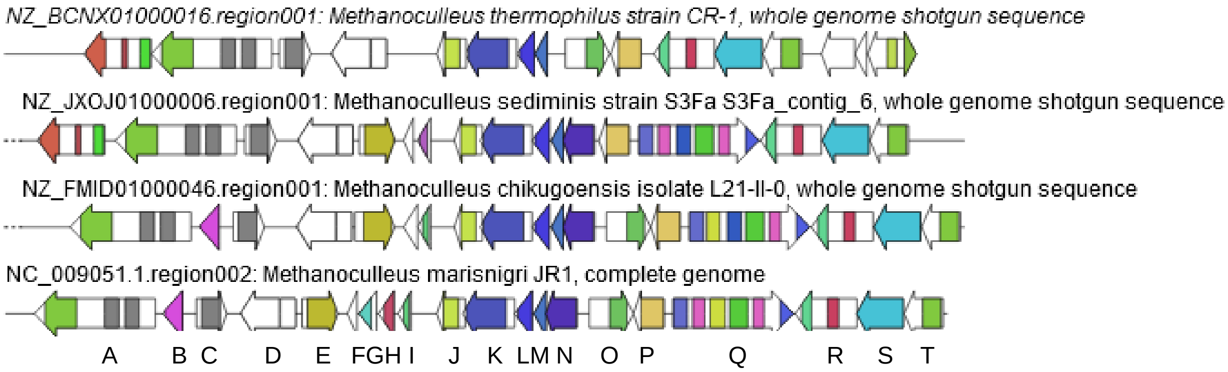

A: excinuclease ABC subunit UvrA
B: ferritin family protein
C: DUF5591 domain-containing protein
D: type III ribulose-bisphosphate carboxylase
E: dienelactone hydrolase family protein
F: hypothetical protein
G: winged helix-turn-helix transcriptional regulator
H: hypothetical protein
I: hypothetical protein
J: TfuA
K: YcaO
L: molybdenum cofactor biosynthesis protein MoaE
M: MoaD/ThiS family protein
N: HesA/MoeB/ThiF family protein
O: GNAT family N-acetyltransferase
P: DUF4935 domain-containing protein
Q: PAS domain S-box protein
R: carboxypeptidase regulatory-like domain-containing protein
S: ABC transporter permease
T: ABC transporter ATP-binding protein

Figure S11. Conserved gene cluster architecture of TfuA - YcaO gene cluster families with most members in Thaumarchaeota *Nitrosocosmicus sp.*. Genes in bold indicate additional biosynthetic or transport genes as annotated by antiSMASH.

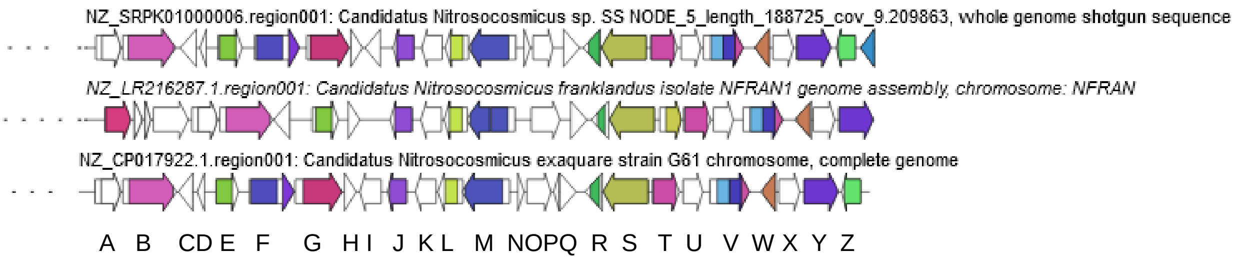

A: ribose 5-phosphate isomerase A
B: amidohydrolase family protein
C: hypothetical protein
D: hypothetical protein
E: ABC transporter ATP-binding protein
F: ABC transporter permease
G: PQQ-dependent sugar dehydrogenase
H: hypothetical protein
I: hypothetical protein
J: YfcE family phosphodiesterase
K: hypothetical protein
L: TfuA
M: YcaO
N: hypothetical protein
O: hypothetical protein
P: hypothetical protein
Q: hypothetical protein
R: 3-isopropylmalate dehydratase small subunit
S: 3-isopropylmalate dehydratase large subunit
T: coenzyme F420-0:L-glutamate ligase
U: hypothetical protein
V: GTP-binding protein
W: reverse transcriptase-like protein
X: hypothetical protein
Y: isocitrate/isopropylmalate dehydrogenase family protein
Z: methyltransferase

Figure S12. Conserved gene cluster architecture of TfuA - YcaO gene cluster families with most members in Cyanobacteria. Genes in bold indicate additional biosynthetic or transport genes as annotated by antiSMASH. Sequence logos of predicted precursor peptides using RiPPER are indicated.

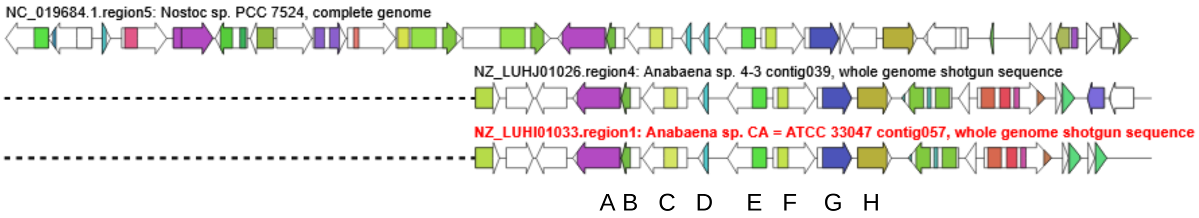

A: tryptophan 7-halogenase
B: 2OG-Fe(II) oxygenase family protein
C: SPASM domain-containing protein
D: precursor peptide (Nif11 family protein)
E: MBL fold metallo-hydrolase
F: TfuA
G: YcaO
H: NAD(P)/FAD-dependent oxidoreductase

Putative precursor peptide 2 (Cluster 275)

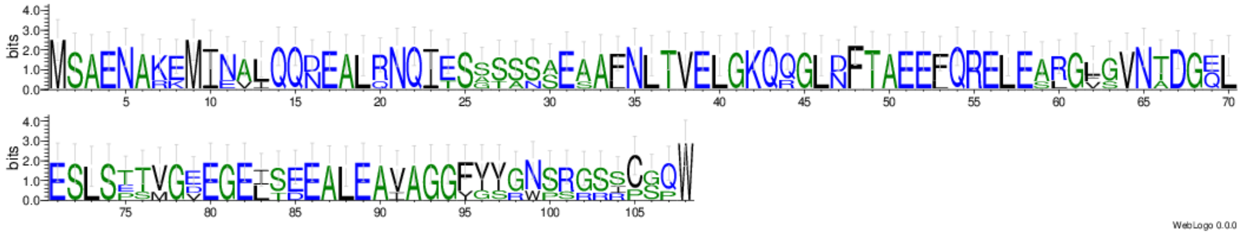

Figure S13. Conserved gene cluster architecture of TfuA - YcaO gene cluster families with most members in Cyanbobacteria *Nostoc sp.*. Genes in bold indicate additional biosynthetic or transport genes as annotated by antiSMASH. Sequence logos of predicted precursor peptides using RiPPER are indicated.

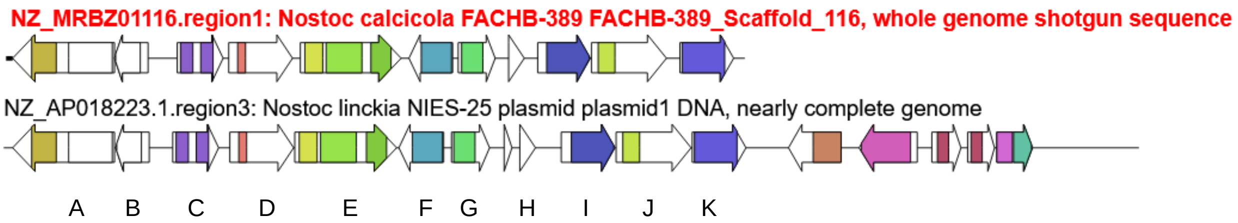

A: adenylate/guanylate cyclase domain-containing protein
B: DUF928 domain-containing protein
C: cyclic nucleotide-binding domain-containing protein
D: NHLP bacteriocin system secretion protein
E: NHLP family bacteriocin export ABC transporter peptidase/permease/ATPase subunit
F: cupin-like domain-containing protein
G: thiopeptide-type bacteriocin biosynthesis protein
H: precursor peptide 1
I: YcaO
J: TfuA
K: cupin-like domain-containing protein

Putative precursor peptide (Cluster 136)

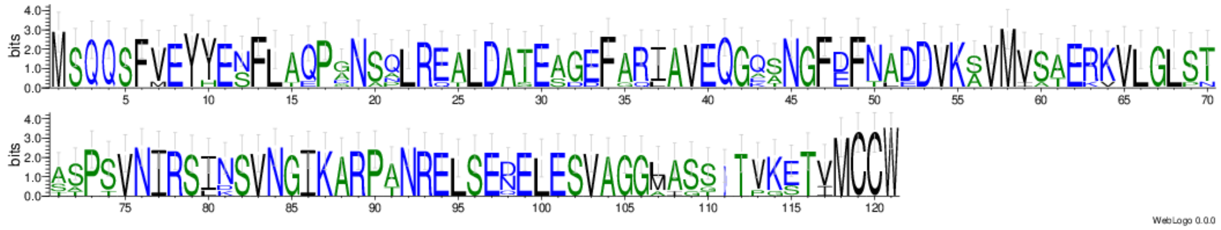

Figure S14. Sequence similarity network of TfuA protein sequences generated using EFI-EST.

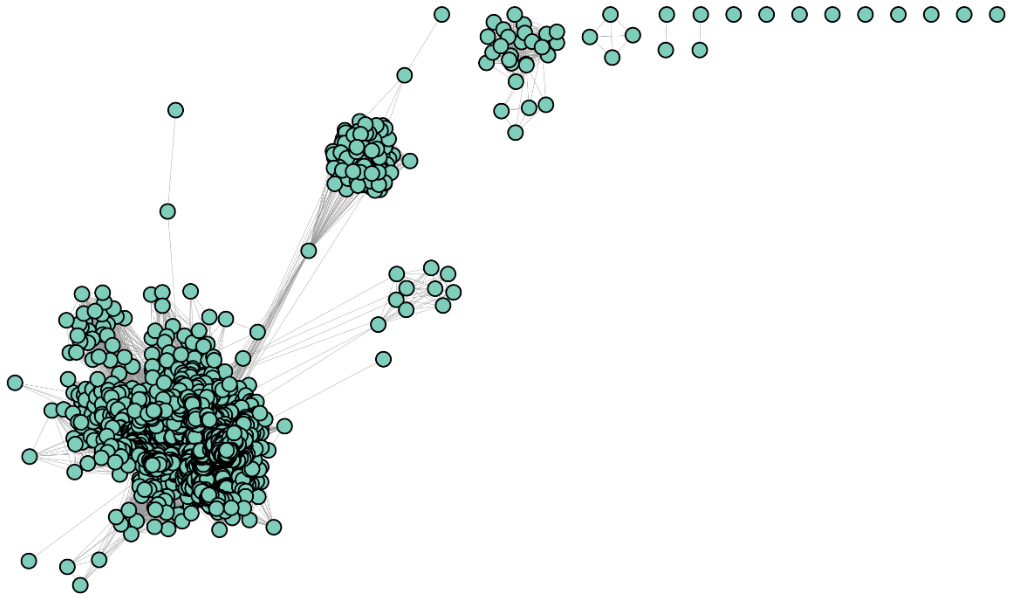

Figure S15. Sequence similarity network of precursor peptides identified through RiPPER within TfuA-related BGCs.

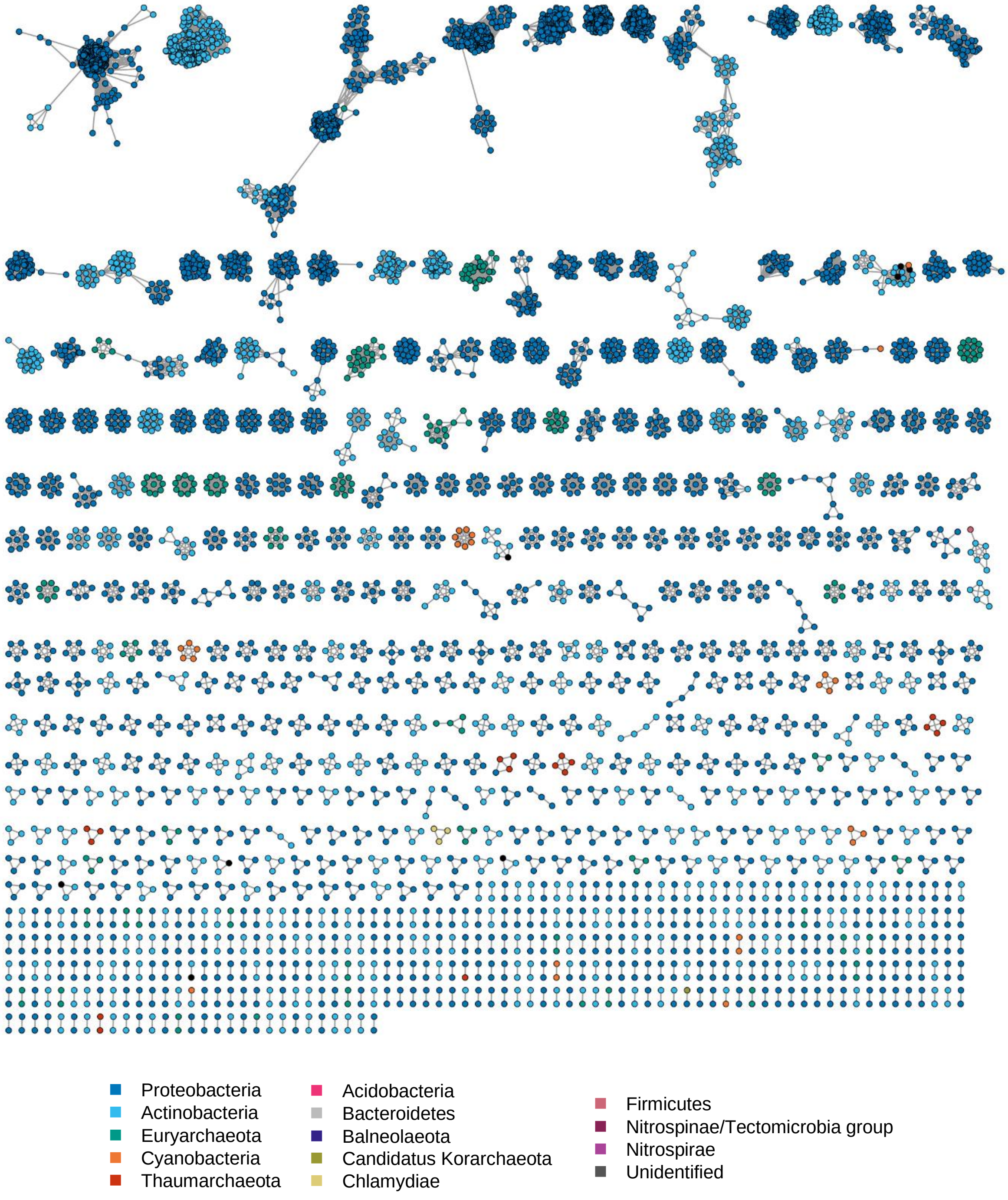

Figure S16. TfuA-related GCFs containing an CCRG-2 family-like precursor peptide as predicted by RiPPER. GCF from *Nostoc sp.* share the same predicted precursor peptide network. Sequence logo of predicted precursor peptide using RiPPER is indicated.

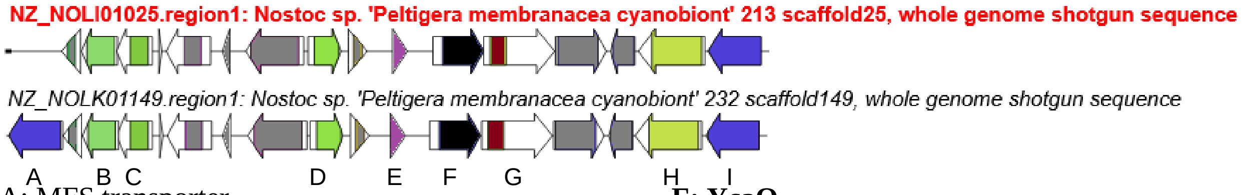

A: MFS transporter
B: multidrug ABC transporter permease
C: multidrug ABC transporter ATP-binding protein
D: isochorismatase
E: putative precursor peptide
F: YcaO
G: TfuA
H: EmrB/QacA family drug resistance transporter
I: MFS transporter

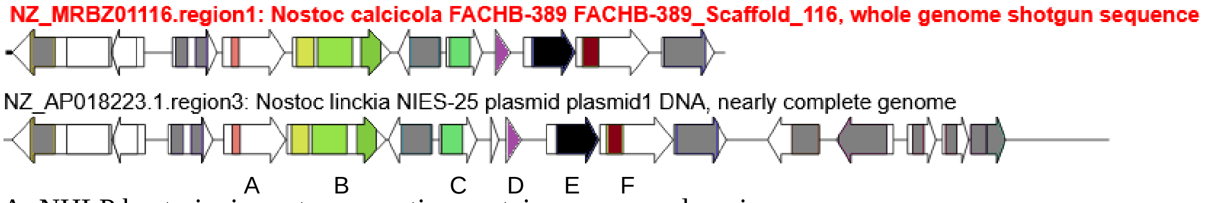

A: NHLP bacteriocin system secretion protein
B: NHLP family bacteriocin export ABC transporter peptidase/permease/ATPase subunit
C: thiopeptide-type bacteriocin biosynthesis protein Lanthipeptide dehydratase, C-terminal domain
D: putative precursor peptide (Cluster 136)
E: YcaO
F: TfuA

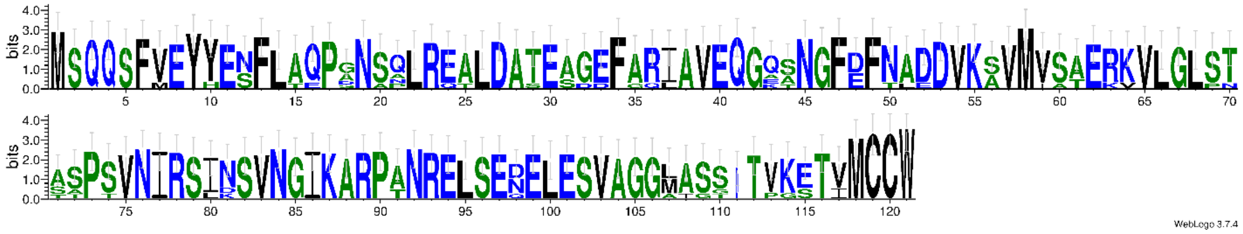

Figure S16 (cont). TfuA-related GCFs containing an CCRG-2 family-like precursor peptide as predicted by RiPPER. GCFs from *Nostoc* share the same predicted precursor peptide network. Sequence logo of predicted precursor peptide using RiPPER is indicated.

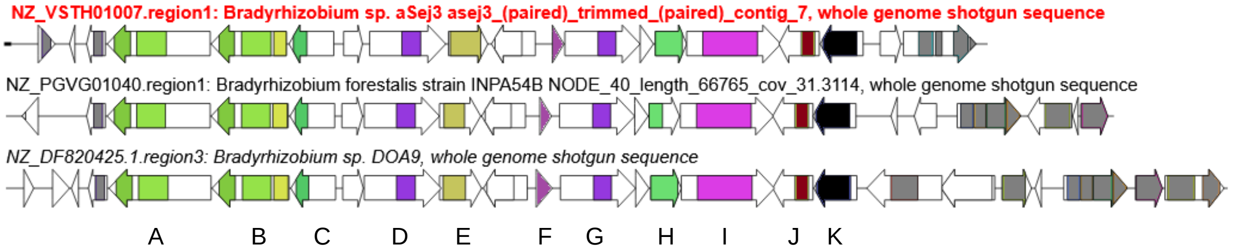

A: NHLP bacteriocin export ABC transporter permease/ATPase subunit
B: NHLP family bacteriocin export ABC transporter peptidase/permease/ATPase subunit
C: NHLP bacteriocin system secretion protein
D: B12-binding domain-containing radical SAM protein
E: indole_PTase
F: putative precursor peptide (Cluster 661)
G: B12-binding domain-containing radical SAM protein
H: Lanthipeptide dehydratase, C-terminal domain
I: Lanthipeptide dehydratase, N-terminal domain
J: TfuA
K: YcaO

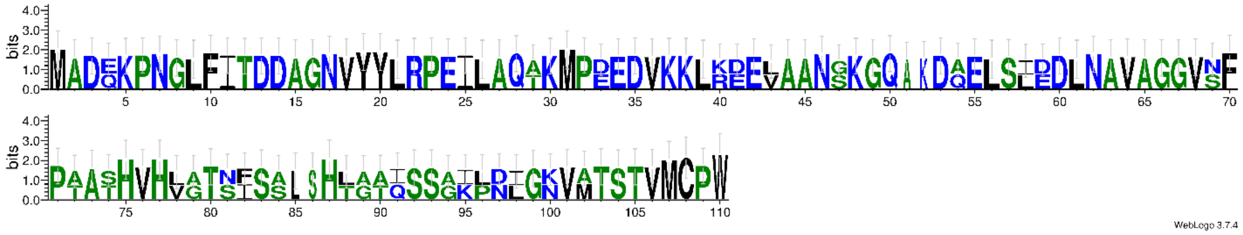

Figure S17. TfuA-related GCFs containing an albusnodin-like precursor peptide as predicted by RiPPER. Sequence logo of predicted precursor peptide using RiPPER is indicated.

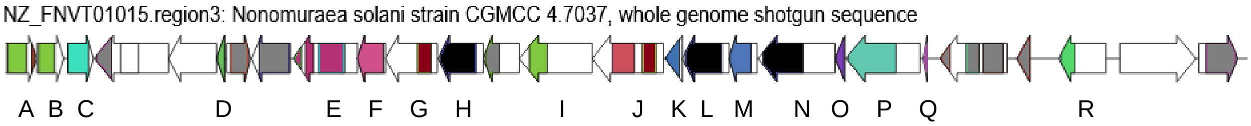

A: branched-chain amino acid transport system ATP-binding protein
B: branched-chain amino acid transport system ATP-binding protein
C: HAD-superfamily hydrolase
D: acyl carrier protein
E: AMP-binding protein
F: thioesterase
G: TfuA
H: YcaO
I: ABC transporter ATP-binding protein
J: protein-L-isoaspartate(D-aspartate) O-methyltransferase with TfuA domain
K: Transglutaminase-like superfamily protein
L: YcaO (ribosomal protein S12 methylthiotransferase accessory factor)
M: SagB-type dehydrogenase domain-containing protein
N: YcaO (thiazole/oxazole-forming peptide maturase, SagD family component)
O: Coenzyme PQQ synthesis protein D (PqqD)
P: albusnodin/ikarugamycin family macrolactam cyclase
Q: putative precursor peptide (Cluster 249)
R: Cytochrome P450

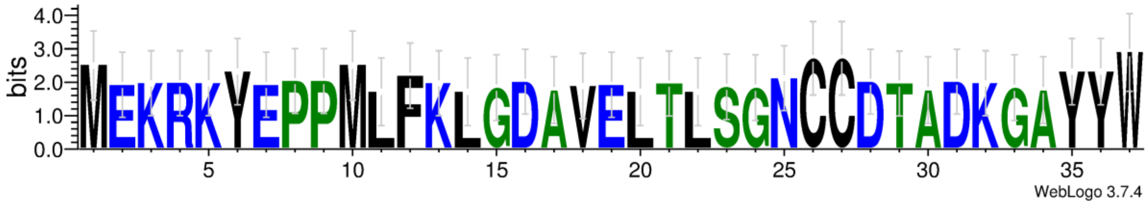

Figure S18. TfuA-related GCFs containing berninamycin-like precursor peptide as predicted by RiPPER. GCFs from *Rhizobium* share the same predicted precursor peptide network. Sequence logo of predicted precursor peptide using RiPPER is indicated.

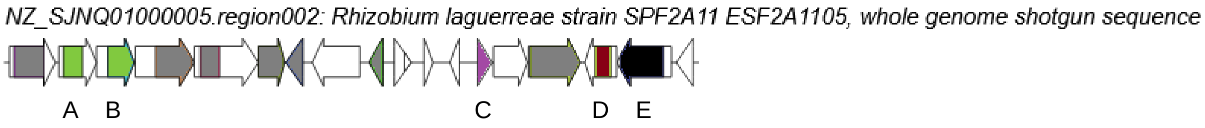

A: ABC transporter permease
B: ABC transporter ATP-binding protein
C: putative precursor peptide (Cluster 288)
D: TfuA
E: YcaO

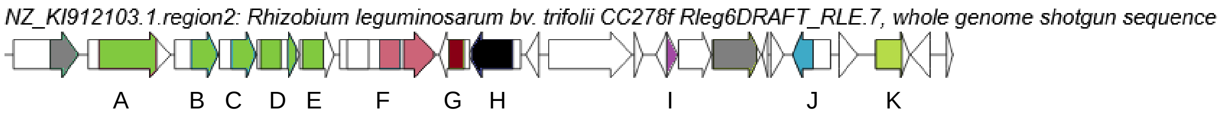

A: ABC transporter substrate-binding protein
B: ABC transporter permease
C: ABC transporter permease
D: ABC transporter ATP-binding protein
E: ABC transporter ATP-binding protein
F: EAL domain-containing protein (diguanylate cyclase)
G: TfuA
H: YcaO
I: putative precursor peptide (Cluster 288)
J: GGDEF domain-containing protein (diguanylate cyclase)
K: SDR family oxidoreductase

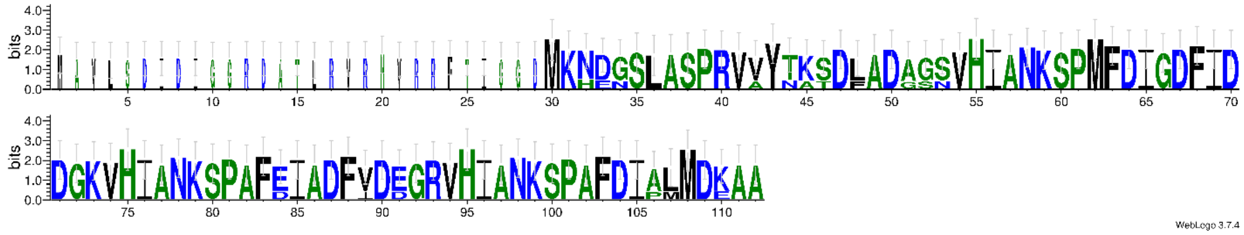

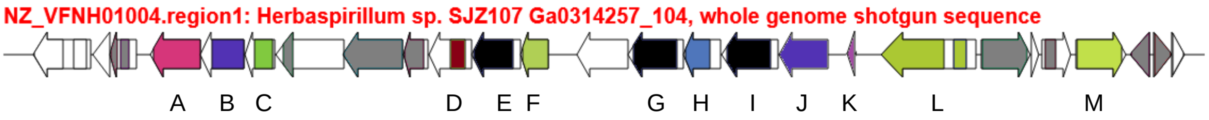

A: aminotransferase class V-fold PLP-dependent enzyme
B: ThiF family adenylyltransferase
C: ABC transporter ATP-binding protein
D: TfuA
E: YcaO
F: ATP-binding cassette domain-containing protein
G: YcaO
H: SagB/ThcOx family dehydrogenase
I: YcaO
J: TOMM precursor leader peptide-binding protein (ThiF)
K: putative precursor peptide (Cluster 486)
L: TonB-dependent receptor
M: multidrug efflux MFS transporter

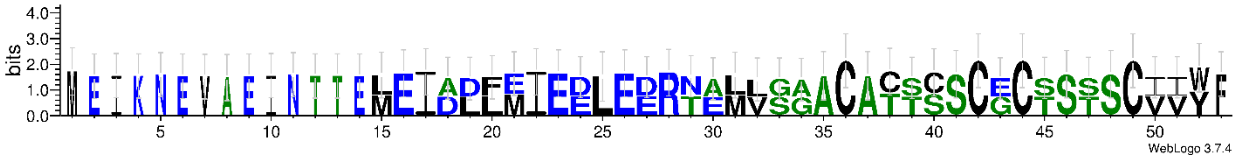

Figure S19. TfuA-related GCF containing with the precursor peptide annotated as a bacteriocin containing thiopeptide-type modifications as predicted by RiPPER. Sequence logo of predicted precursor peptide using RiPPER is indicated.

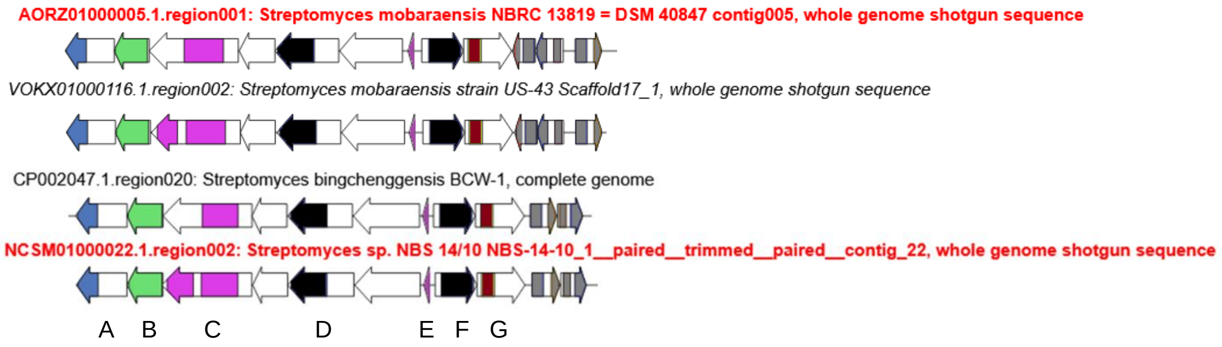

A: nitroreductase (PF00881)
B: Lanthipeptide dehydratase, C-terminal domain
C: Lanthipeptide dehydratase, N-terminal domain
D: YcaO
E: putative precursor peptide (Cluster 91)
F: YcaO
G: TfuA

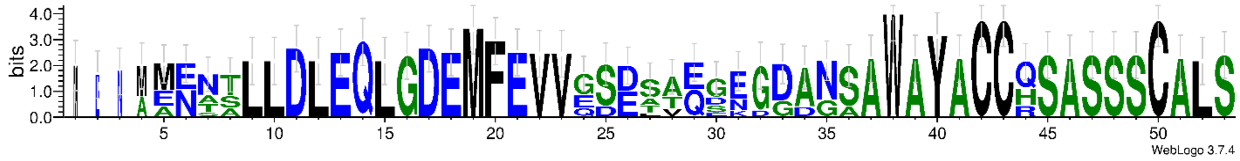

Figure S20. TfuA-related GCFs containing Nif-like precursor peptides as predicted by RiPPER.

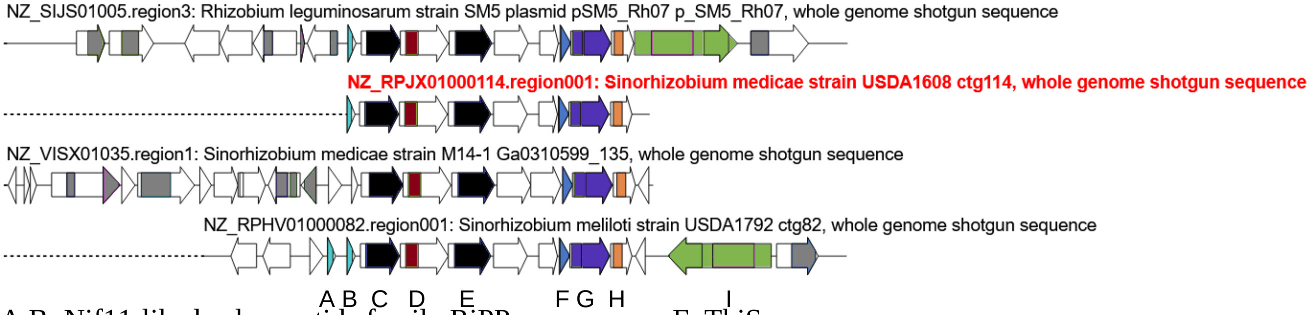

A,B: Nif11-like leader peptide family RiPP precursor
C: YcaO
D: TfuA
E: YcaO
F: ThiS
G: ThiF molybdopterin-synthase adenylyltransferase MoeB
H: class I SAM-dependent methyltransferase
I: type 2 lantipeptide synthetase LanM

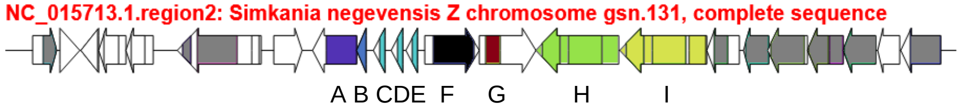

A: ThiF putative adenylyltransferase/sulfurtransferase MoeZ
B: ThiS
C,D,E: Nif11-like leader peptide family RiPP precursor
F: YcaO
G: TfuA
H: ATP-binding cassette domain-containing protein
I: Peptidase C39-containing ATP-binding cassette domain-containing protein

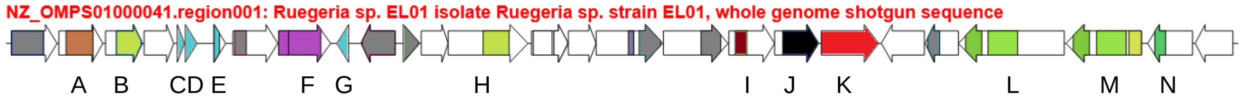

A: extracellular solute-binding protein (sugar-binding lipoprotein)
B: sugar ABC transporter permease
C, D, E, G: Nif11-like leader peptide family RiPP precursor
F: tryptophan 7-halogenase
H: MFS transporter
I: TfuA
J: YcaO
K: proteusin epimerase (RRE)
L: NHLP bacteriocin export ABC transporter permease/ATPase subunit
M: NHLP family bacteriocin export ABC transporter peptidase/permease/ATPase subunit
N: NHLP bacteriocin system secretion protein

Figure S21. Frequencies of additional modification genes (A) and other genes (B) as annotated by antiSMASH found within TfuA-related BGCs.

A

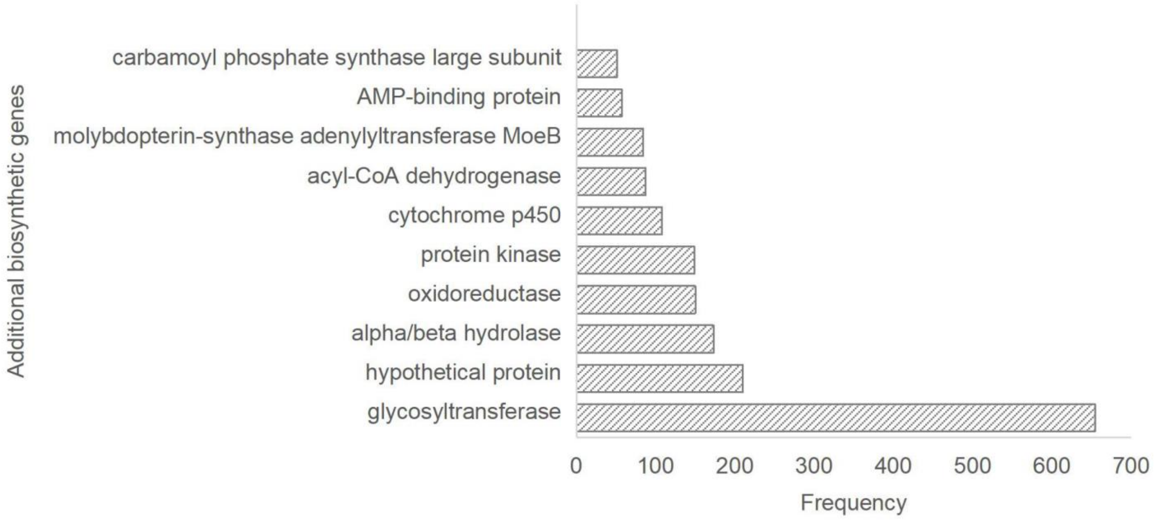

B

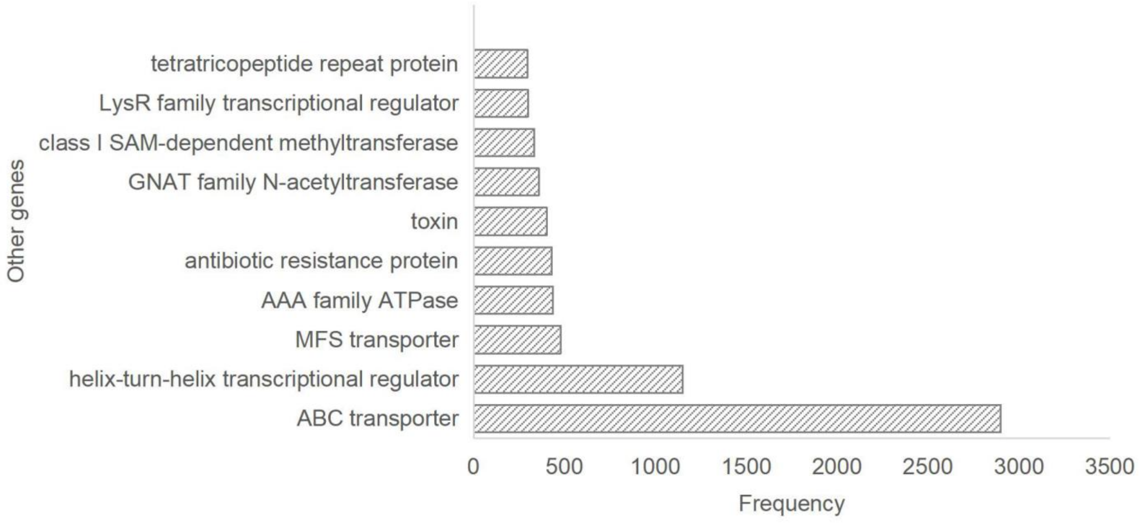

Figure S22. TfuA-related GCF containing an iron-containing redox enzyme family protein. Sequence logo of predicted precursor peptide using RiPPER is indicated.

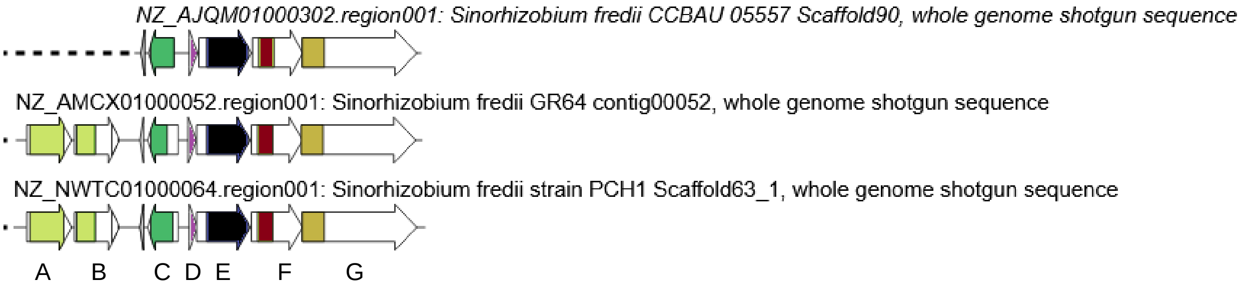

A: sulfate ABC transporter substrate-binding protein
B: sulfate/molybdate ABC transporter ATP-binding protein
C: iron-containing redox enzyme family protein
D: putative precursor peptide (Cluster 33)
E: YcaO
F: TfuA
G: adenylate/guanylate cyclase domain-containing protein

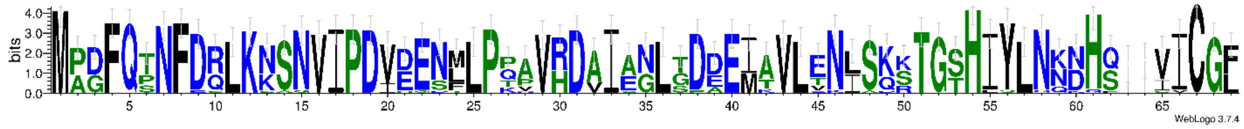

Figure S23. TfuA-related GCF containing a fused *tfuA* and *ycaO* genes. Sequence logo of predicted precursor peptide using RiPPER is indicated.

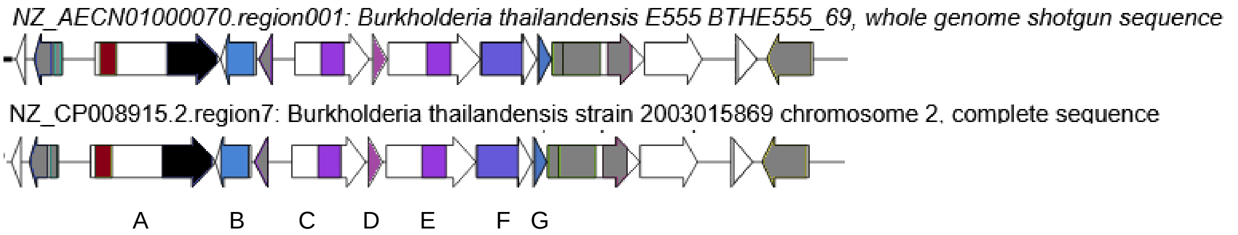

A: Fused TfuA-Ycao
B: phytanoyl-CoA dioxygenase family protein
C: radical SAM protein
D: putative precursor peptide (Cluster 127)
E: RiPP maturation radical SAM C-methyltransferase
F: cupin-like protein
G: ThiS

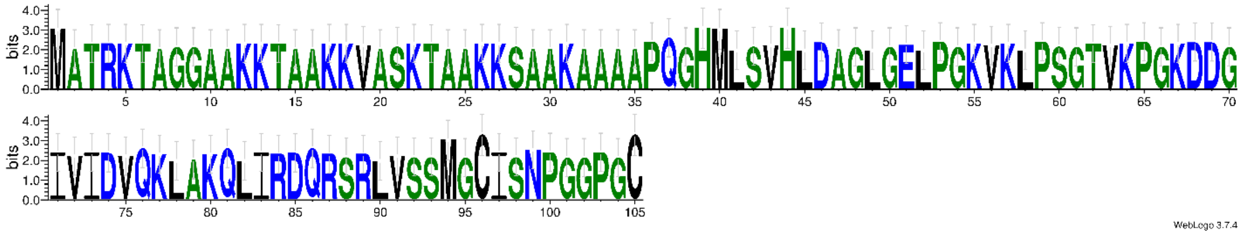

Figure S24. TfuA-related GCF annotated to also produce ranthipeptides as annotated by antiSMASH. Both GCFs share the same precursor peptide network. Sequence logo of predicted precursor peptide using RiPPER is indicated.

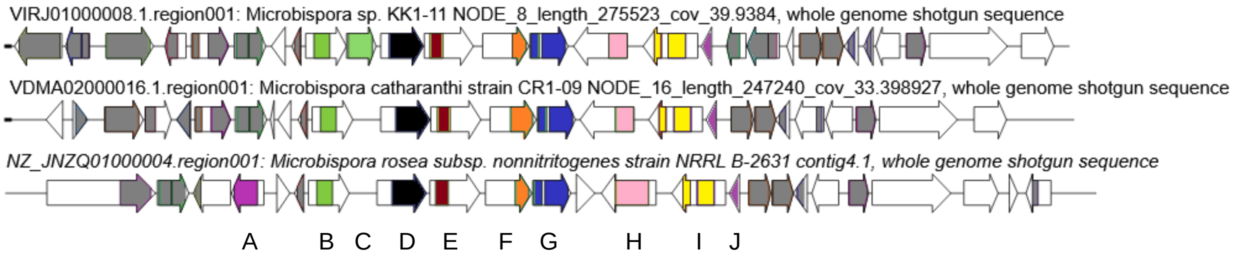

A: alpha/beta hydrolase
B: ABC transporter ATP-binding protein
C: ABC transporter permease
D: YcaO
E: TfuA
F: cytochrome P450
G: O-methyltransferase
H: FAD-dependent monooxygenase
I: radical SAM protein (ranthipeptide)
J: putative precursor peptide (Cluster 14)

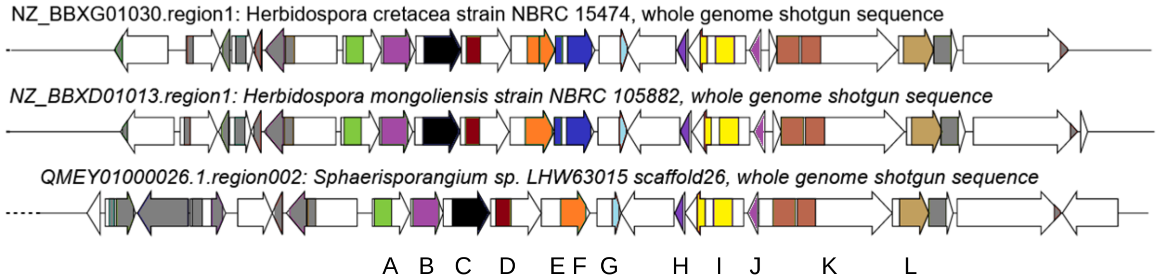

A: ABC transporter ATP-binding protein
B: ABC transporter permease
C: YcaO
D: TfuA
E: cytochrome P450
F: O-methyltransferase
G: LuxR family DNA-binding response regulator
H: PqqD family protein (Stand_Alone_Lasso_RRE PF05402)
I: radical SAM protein (ranthipeptide)
J: putative precursor peptide (Cluster 14)
K: adenylate/guanylate cyclase domain-containing protein
L: S8 family serine peptidase

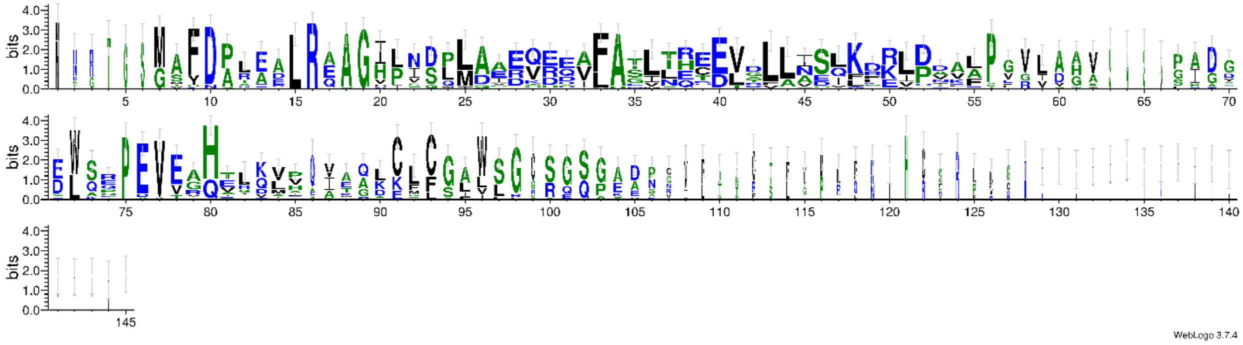

Figure S25. TfuA-related GCF annotated to also produce linear azole-containing peptides as annotated by antiSMASH. Sequence logo of predicted precursor peptide using RiPPER is indicated.

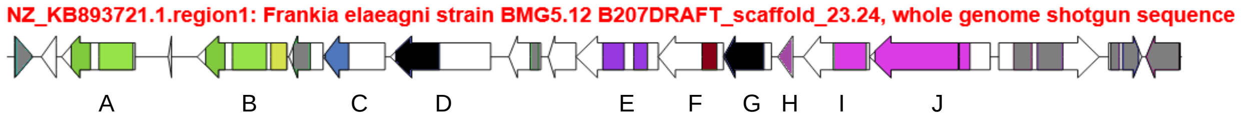

A: ATP-binding cassette domain-containing protein
B: NHLP family bacteriocin export ABC transporter peptidase/permease/ATPase subunit
C: SagB/ThcOx family dehydrogenase
D: YcaO (TOMM precursor leader peptide-binding protein)
E: RiPP maturation radical SAM protein 1
F: TfuA
G: YcaO
H: putative precursor peptide (Cluster 609)
I: Lanthipeptide dehydratase, C-terminal domain
J: Lanthipeptide dehydratase, N-terminal domain

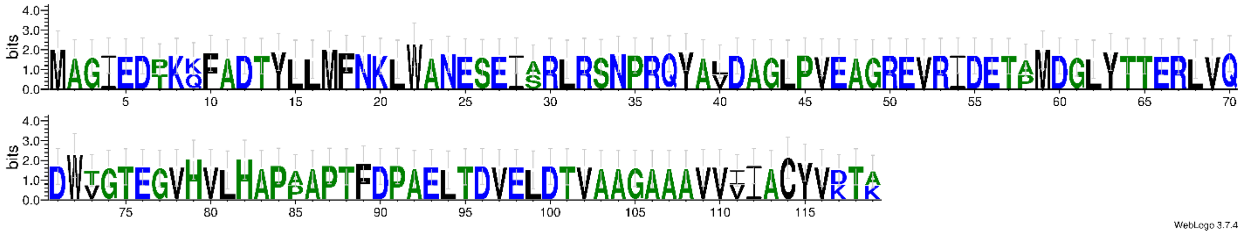

Figure S26. TfuA-related GCFs annotated to also produce thiopeptides as annotated by antiSMASH. Sequence logo of predicted precursor peptide using RiPPER is indicated.

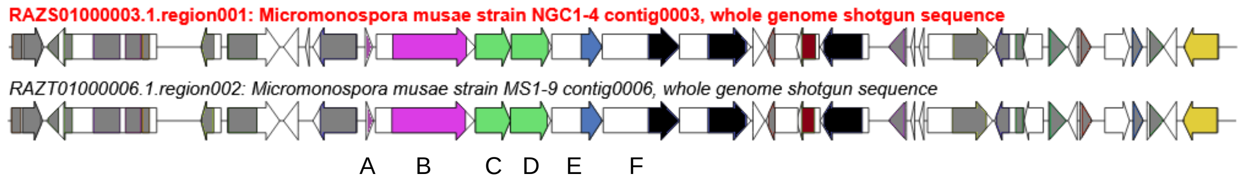

A: putative precursor peptide (Cluster 192)
B: Lanthipeptide dehydratase, N-terminal domain
C: Lanthipeptide dehydratase, C-terminal domain
D: Lanthipeptide dehydratase, C-terminal domain
E: SagB/ThcOx family dehydrogenase
F: YcaO (heterocycloanthracin_C_RRE)
G: YcaO
H: TfuA
I: YcaO
J: aldo/keto reductase family oxidoreductase

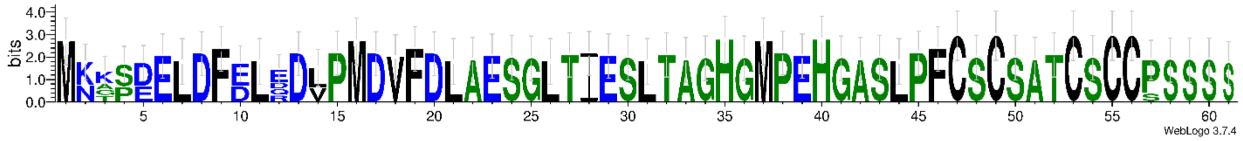

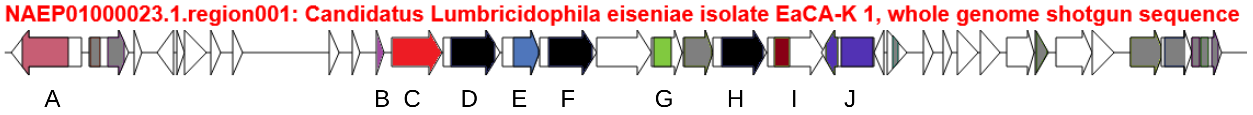

A: ABC transporter substrate-binding protein
B: putative precursor peptide (Cluster 486)
C: ThiF Goadsporin_CD_RRE
D: YcaO
E: nitroreductase (PF00881)
F: YcaO
G: ABC transporter ATP-binding protein
H: YcaO
I: TfuA
J: ThiF

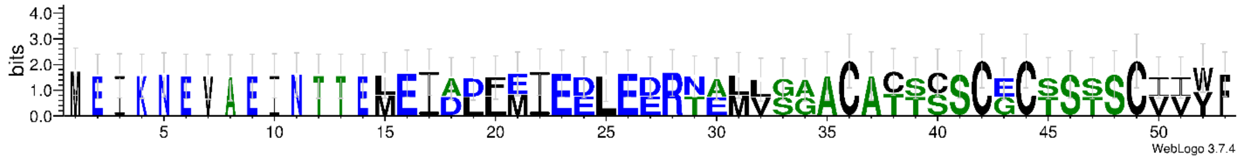

Figure S26 (cont.). TfuA-related GCFs annotated to also produce thiopeptides as annotated by antiSMASH. Sequence logo of predicted precursor peptide using RiPPER is indicated.

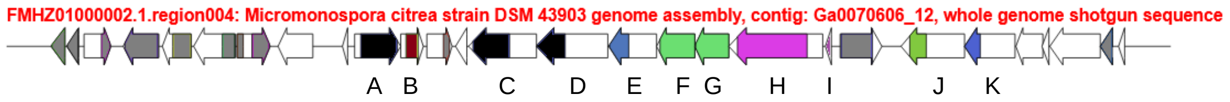

A: YcaO
B: TfuA
C: YcaO (ribosomal protein S12 methylthiotransferase accessory factor)
D: YcaO
E: SagB-type dehydrogenase domain-containing protein
F: Lantibiotic biosynthesis dehydratase C-term
G: Lantibiotic biosynthesis dehydratase C-term
H: Lanthipeptide dehydratase, N-terminal domain
I: precursor peptide (Cluster 192)
J: Alpha/beta hydrolase family protein (Peptidase_S9)
K: dehydrogenase

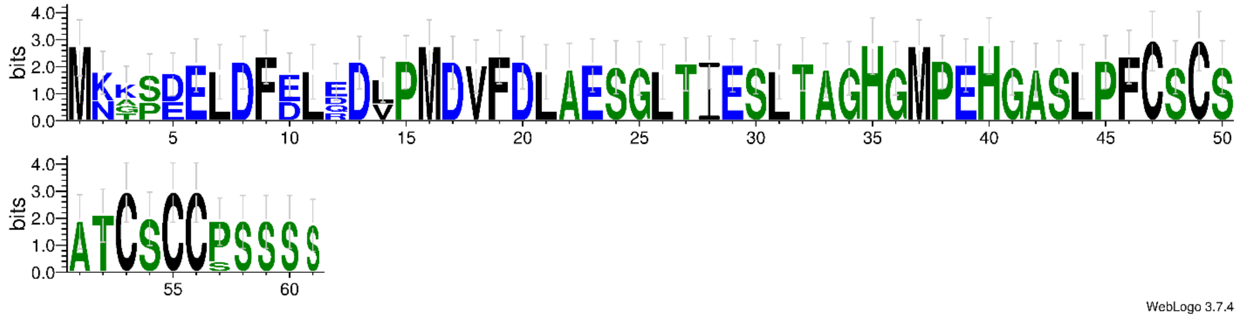

Figure S27. TfuA-related GCFs containing multiple *ycaO* genes without further annotation. Sequence logo of predicted precursor peptide using RiPPER is indicated.

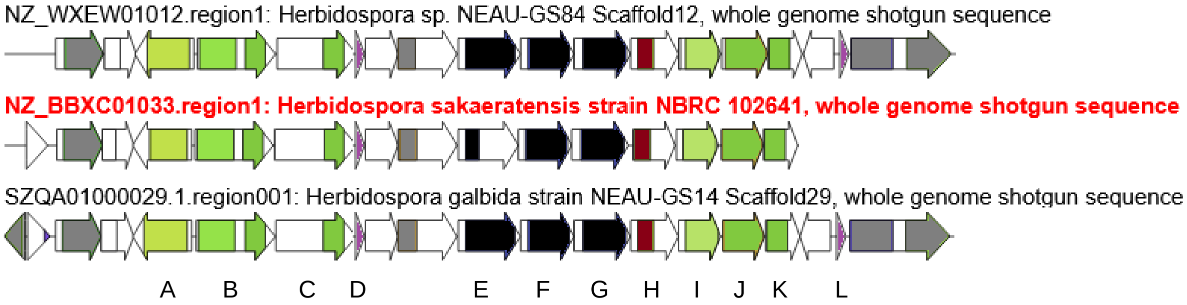

A: MFS transporter
B: ABC transporter ATP-binding protein
C: ABC transporter ATP-binding protein
D: putative precursor peptide (Cluster 200)
E, F, G: YcaO
H: TfuA
I: iron compound ABC transporter, periplasmic
J: iron ABC transporter permease
K: ABC transporter ATP-binding protein
L: putative precursor peptide 2

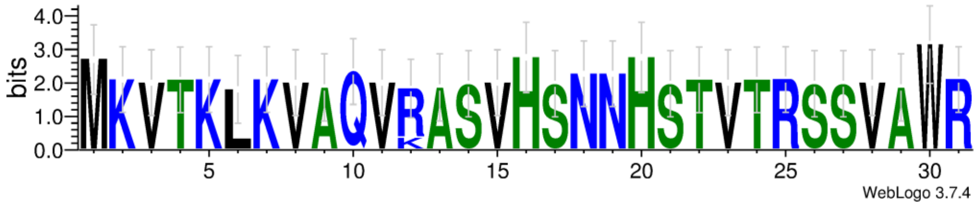

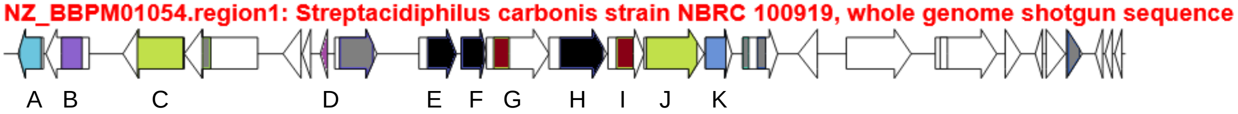

A: flavin reductase family protein
B: alpha/beta hydrolase (Peptidase S9)
C: MFS transporter
D: putative precursor peptide (Cluster 515)
E, F: YcaO
G: TfuA
H: YcaO
I: TfuA
J: MFS transporter
K: GNAT family N-acetyltransferase

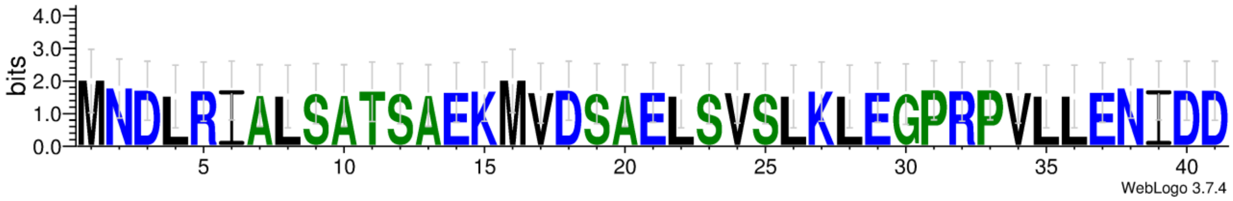

Figure S27 (cont.). TfuA-related GCFs containing multiple *ycaO* genes without further annotation. Sequence logo of predicted precursor peptide using RiPPER is indicated.

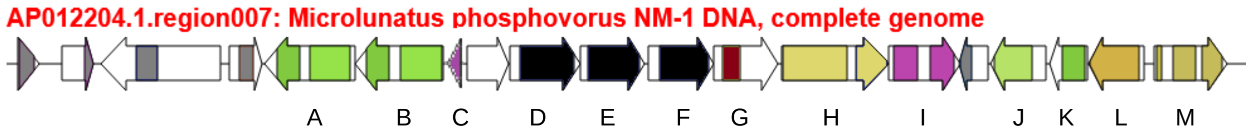

A: ABC transporter permease/ATP-binding protein
B: ABC transporter permease/ATP-binding protein
C: putative precursor peptide (Cluster 224)
D, E, F: YcaO
G: TfuA
H: prolyl endopeptidase (Peptidase S9)
I: MatE efflux family protein
J: ABC transporter substrate-binding protein
K: ABC transporter ATP-binding protein
L: ABC transporter permease protein

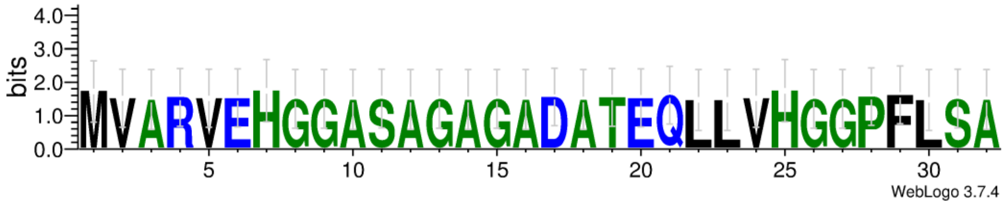

Figure S28. Sequence logo of the ATP-binding sequence motifs identified from TfuA-associated YcaO proteins.

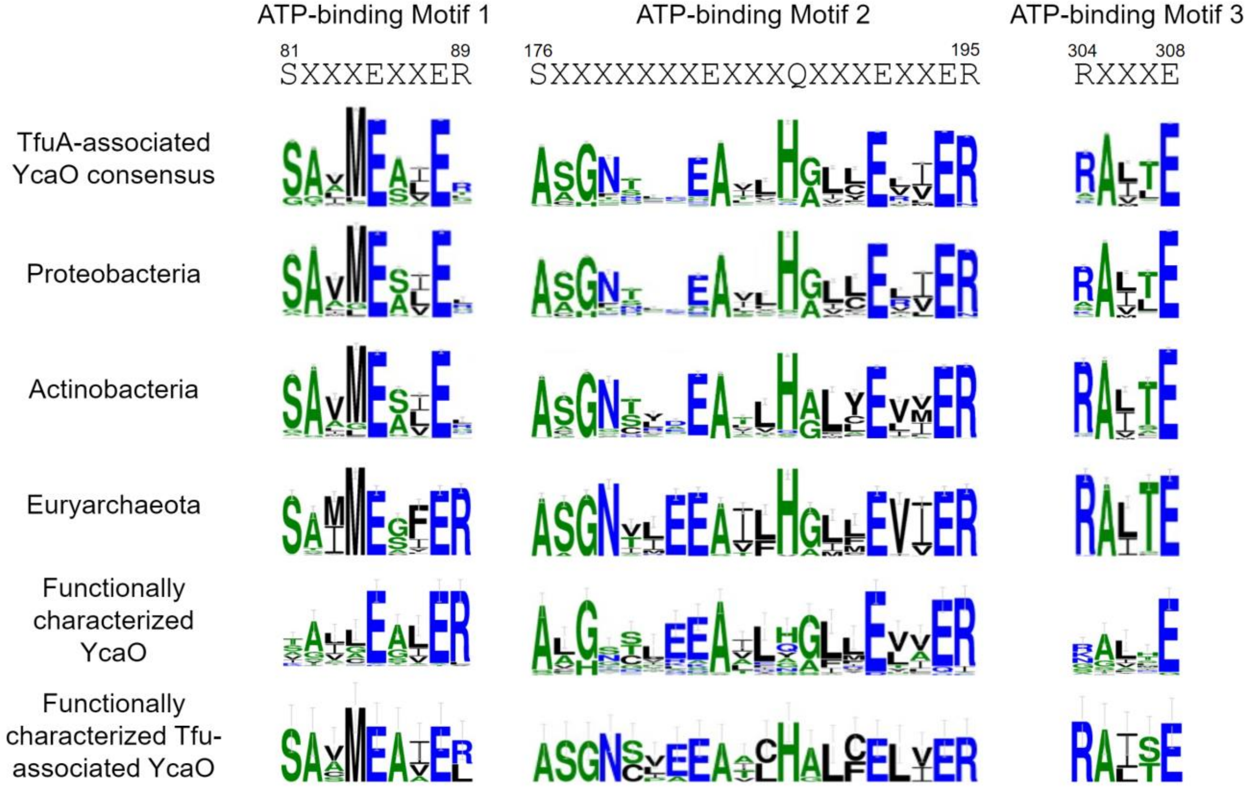

Supplement: Supplementary file 2 [file Data_Sheet_1.PDF]
